# Coal dust nanoparticles induced pulmonary fibrosis by promoting inflammation and epithelial-mesenchymal transition via the NF-κB/NLRP3 pathway driven by IGF1/ROS-mediated AKT/GSK3β signals

**DOI:** 10.1038/s41420-022-01291-z

**Published:** 2022-12-29

**Authors:** Yinci Zhang, Jiaojiao Liang, Niandie Cao, Jiafeng Gao, Li Song, Xiaolong Tang

**Affiliations:** 1 Joint National-Local Engineering Research Centre for Safe and Precise Coal Mining (Anhui University of Science and Technology), Huainan, 232001 China; 2 Institute of Environment-friendly Materials and Occupational Health of Anhui University of Science and Technology, Wuhu, 241003 China; 3 Medcial School, Anhui University of Science & Technology, Huainan, 232001 China

**Keywords:** Cell signalling, Predictive markers

## Abstract

Pneumoconiosis is the most common and serious disease among coal miners. In earlier work on this subject, we documented that coal dust (CD) nanoparticles (CD-NPs) induced pulmonary fibrosis (PF) more profoundly than did CD micron particles (CD-MPs), but the mechanism has not been thoroughly studied. Based on the GEO database, jveen, STRING, and Cytoscape tools were used to screen hub genes regulating PF. Particle size distribution of CD were analyzed with Malvern nanoparticle size potentiometer. Combining 8 computational methods, we found that IGF1, POSTN, MMP7, ASPN, and CXCL14 may act as hub genes regulating PF. Based on the high score of IGF1 and its important regulatory role in various tissue fibrosis, we selected it as the target gene in this study. Activation of the IGF1/IGF1R axis promoted CD-NPs-induced PF, and inhibition of the axis activation had the opposite effect in vitro and in vivo. Furthermore, activation of the IGF1/IGF1R axis induced generation of reactive oxygen species (ROS) to promote epithelial-mesenchymal transition (EMT) in alveolar epithelial cells (AECs) to accelerate PF. High-throughput gene sequencing based on lung tissue suggested that cytokine-cytokine receptor interaction and the NF-kB signaling pathway play a key role in PF. Also, ROS induced inflammation and EMT by the activation of the NF-kB/NLRP3 axis to accelerate PF. ROS can induce the activation of AKT/GSK3β signaling, and inhibition of it can inhibit ROS-induced inflammation and EMT by the NF-kB/NLRP3 axis, thereby inhibiting PF. CD-NPs induced PF by promoting inflammation and EMT via the NF-κB/NLRP3 pathway driven by IGF1/ROS-mediated AKT/GSK3β signals. This study provides a valuable experimental basis for the prevention and treatment of coal workers’ pneumoconiosis.

**Figure:**
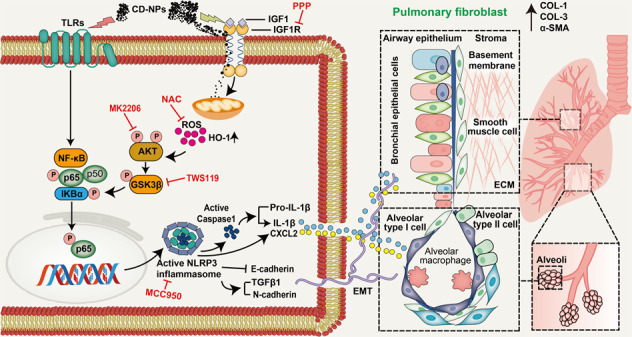
Illustration of the overall research idea of this study: IGF1 stimulates coal dust nanoparticles induced pulmonary fibrosis by promoting inflammation and EMT via the NF-κB/NLRP3 pathway driven by ROS-mediated AKT/GSK3β signals.

Illustration of the overall research idea of this study: IGF1 stimulates coal dust nanoparticles induced pulmonary fibrosis by promoting inflammation and EMT via the NF-κB/NLRP3 pathway driven by ROS-mediated AKT/GSK3β signals.

## Introduction

Coal is an important energy source in the world, and it will remain the main energy source for economic development for a long time [1, 2]. Pneumoconiosis is a group of the most common and serious occupational diseases among coal miners, mainly characterized by diffuse fibrosis of the lung tissue [3–5]. Although countries around the world have taken measures to prevent and control pneumoconiosis among coal miners, it has made a comeback in recent years [6]. The reaction rate, settling and solubility of respirable coal dust (CD) are important factors in its ability to induce lung injury and can be directly influenced by its particle size [7]. Therefore, CD with different particle sizes may have different degrees of impact on human health. Extensive literature suggests that nanoparticles are highly pathogenic due to their unique physicochemical properties [8–10]. Our previous work [11] revealed that CD nanoparticles (CD-NPs) more seriously induce pulmonary fibrosis (PF) than do CD micron particles (CD-MPs), but the mechanism needs further exploration.

During the formation of PF, differentiation of lung fibroblasts into myofibroblasts cooperates with the upregulation of α-smooth muscle actin (α-SMA) and the synthesis of collagens to form the extracellular matrix (ECM) [12]. Abnormal repair of lung tissue is often due to excessive collagen and ECM production by lung epithelial cells [13]. Initial studies have revealed that the persistent effects of chronic inflammation led to the formation of PF [14, 15]. Subsequent studies have found that particulate matter induces epithelial-mesenchymal transition (EMT) in alveolar epithelial cells (AECs) after lung injury, which triggers PF [16, 17]. In parallel, inflammasomes can respond to lung injury [18]. These actions also mean that excessive EMT and unbalanced inflammation can co-exist and promote the progression of idiopathic PF.

It has been reported that PM2.5 can directly induce lung epithelium damage [19]. Injured lung epithelial cells can exhibit a myofibroblastic phenotype by EMT, which contributes to abnormal wound healing and fibrosis [20–22]. During EMT, E-cadherin decreases in epithelial cells and N-cadherin increases in mesenchymal cells [23]. TGF-β1 reportedly plays an important role in disease progression by promoting EMT [23–25]. In addition, TGF-β1 has been identified as an important profibrotic factor that induces EMT in PF [26–28], and EMT promotes myofibroblast activation and ECM production [20–22]. NLRP3 is one of the best-characterized inflammasomes [29, 30]. Intracellular reactive oxygen species (ROS), triggered by foreign substances can activate NF-κB signaling, causing phosphorylation and translocation of NF-κB p65, resulting in transcriptional upregulation of inflammasome-related components (NLRP3, pro-IL-1β, and pro-IL-18), which in turn activate caspase-1 [31, 32]. Activated caspase-1 can catalyze the maturation of the proinflammatory cytokines IL-1β and IL-18, thereby amplifying the inflammatory response [31]. Inflammatory chemokines, such as CXCL2, are also recruited by activation of the NLRP3 inflammasome [33, 34]. Recent studies describe the induction of NLRP3 inflammasome activation and PF by fine particulate matter in the air. Furthermore, inhibiting NLRP3 inflammasome activation can slow the progression of various diseases by inhibiting inflammation and/or EMT [35–37].

AKT/GSK3β is an important transduction signal in cells, and GSK3β is an important AKT signaling substrate, which has a regulatory effect on mitochondrial activity [38]. Phosphorylation at Ser9 by Ser/Thr protein kinase inhibits GSK3β activation [38]. The AKT/GSK3β signaling pathway can be involved in the progression of PF through the EMT pathway [39, 40]. Yang et al. [41] proposed that nickel (II) ions exacerbate bleomycin-induced lung inflammation and fibrosis by activating the ROS/AKT signaling pathway. Previous studies have also shown that the NF-κB signaling pathway can be activated by AKT activation and GSK3β inactivation [42, 43]. Taken together, these findings suggest that NF-κB/NLRP3 signaling may be driven by ROS/AKT/GSK3β signaling to promote PF.

High-throughput microarray platforms are useful tools for detecting genetic alterations in many diseases [44]. We identified five hub genes -- insulin-like growth factor 1 (IGF1), periostin (POSTN), matrix metalloproteinase 7 (MMP7), asporin (ASPN) and chemokine (C-X-C motif) ligand-14 (CXCL14) -- that regulate PF progression. Among various profibrotic cytokines, IGF1 has been shown to promote fibroblast proliferation, migration, and collagen production [45]. Pharmacological inhibition of IGF1 signaling and its downstream pathways may have critical therapeutic potential in the treatment of PF [46]. The biological function of IGF1 is completed by binding to its membrane receptor IGF-1R. In animal models, blocking the IGF1 pathway with an anti-IGF-IR monoclonal antibody attenuated bleomycin-induced lung injury [47]. Reports have shown that IGF1 is also associated with the generation of ROS [48, 49]. Therefore, combining the scores of central genes by the eight algorithms in this study, we focused on the role of the IGF1/IGF1R signaling in CD-NPs-induced PF and its relationship among with ROS generation, NF-κB/NLRP3 signaling and AKT/GSK3β signaling.

We hypothesized that CD-NPs stimulation of AECs caused increased ROS production by activating IGF1/IGF1R signaling, which then activates AKT/GSK3β signaling to further induce NF-κB/NLRP3 signaling-mediated inflammation and EMT to promote PF. In this study, we investigated the roles and interrelationships of the IGF1/IGF1R signaling, ROS generation, AKT/GSK3β signaling, and NF-κB/NLRP3 signaling in CD-NPs-induced PF, thereby elucidating the potential mechanism of CD-NPs-induced PF. This study provides a valuable reference for the prevention and treatment of coal workers’ pneumoconiosis.

## Results

### IGF1 identified as a hub gene in PF

**Fig. 1:**
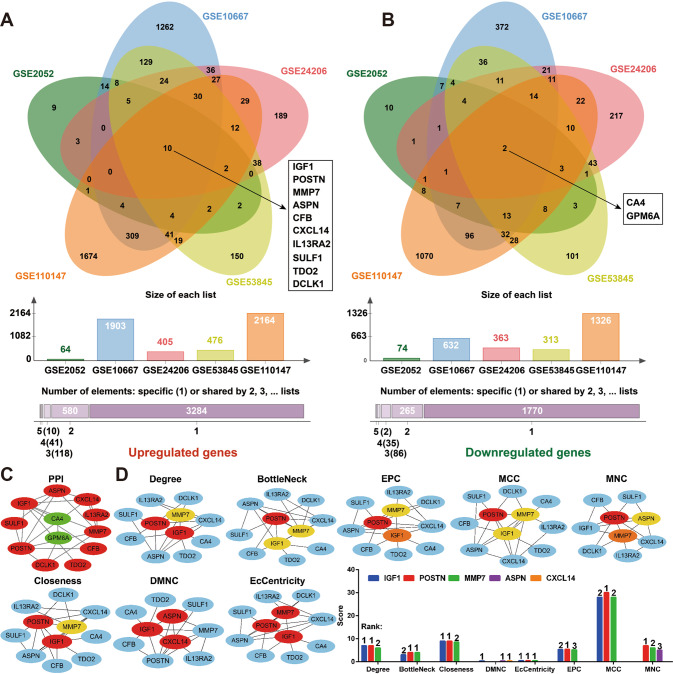
IGF1 identified as a hub gene in PF. **A**, **B** The up- and down-regulated intersection genes among the 5 datasets were selected by jvenn tool. **C** The protein–protein interaction (PPI) network analysis of the up- and down-regulated intersection genes was performed by STRING and Cytoscape software. **D** The hub genes were predicted by the plug-in cytoHubba of Cytoscape software and the top three nodes ranked by MCC, DMNC, MNC, Degree, EPC, BottleNeck, EcCentricity and Closeness.

GSE2052, GSE10667, GSE24206, GSE53845, and GSE110147 were selected for differentially expressed genes analysis in PF. Among them, GSE2052 contained 13 PF tissue specimens and 11 normal tissue specimens; GSE10667 contained 8 PF tissue specimens and 15 normal tissue specimens; GSE24206 contained 17 PF tissue specimens and 6 normal tissue specimens; GSE53845 contained 40 PF tissue specimens and 8 normal tissue specimens; and GSE110147 contained 22 PF tissue specimens and 11 normal tissue specimens. As illustrated in Fig. 1A, B, IGF1, POSTN, MMP7, ASPN, CFB, CXCL14, IL13RA2, SULF1, TDO2, and DCLK1 were selected as upregulated intersection genes in the 5 datasets, and CA4 and GPM6A were selected as down-regulated intersection genes. The PPI enrichment *p*-value of the above 12 genes is 5.84e−07, and a total of 12 nodes and 22 edges are present in the PPI network (Fig. 1C). IGF1, POSTN, MMP7, ASPN, and CXCL14 were preliminarily selected as hub genes of PF by MCC, DMNC, MNC, Degree, EPC, BottleNeck, EcCentricity and Closeness 8 algorithms (Fig. 1D). Based on IGF1’s score in 8 algorithms, the number of times it was selected as a hub gene, and its role in PF through literature analysis, we classified it as a target gene.

### IGF1/IGF1R signaling is significantly activated in models of pre-fibrosis in vitro and in vivo

**Fig. 2:**
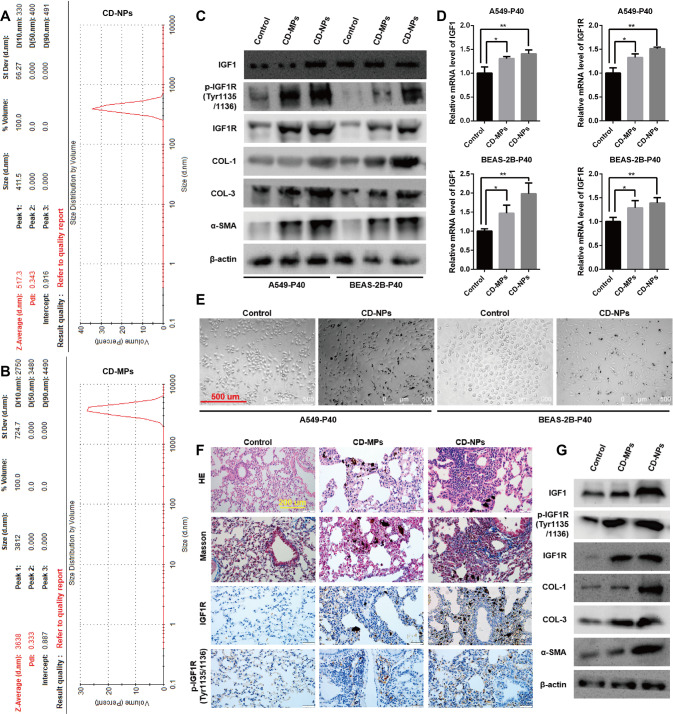
IGF1/IGF1R signaling is significantly activated in models of pre-fibrosis in vitro and in vivo. **A**, **B** The size distribution and polydispersity index (PDI) of CD-MPs and CD-NPs were measured with a Malvern Nanoparticle Size Potentiometer. **C**, **D** IGF1/IGF1R signaling was detected by western blot and qRT-PCR in models of pre-fibrosis in vitro. **E** Cell morphology in an in vitro pre-fibrosis model was examined by inverted microscope. **F**, **G** IGF1/IGF1R signaling was detected by immunohistochemistry and western blot in models of pre-fibrosis in vivo.

Determination of particle size distribution revealed that 59% of the CD-NPs were smaller than 500 nm, and all were smaller than 1 µm (Fig. 2A). 86% of the CD-MPs were larger than 5 µm (Fig. 2B). However, the PDI values of CD-NPs (PDI = 0.343) and CD-MPs (PDI = 0.333) were not significantly different (Fig. 2A, B). The Zeta potentials of CD-NPs (20.9 mV) and CD-MPs (−18.6 mV) were both in the range of ±10 mV to ±30 mV, suggesting that both CD particles have certain adhesion and aggregation properties (Fig. S1). The two CD particles mainly contain C, N, O, Al and Si, with the CD-NPs having higher C content than the CD-MPs, and the opposite content of O, Al and Si (Fig. S2). Data from a non-replicated parallel assay of the above results have been published in the *Journal of Respiratory Research* [11].

Based on previous methods and standard parameters, we established in vivo and in vitro mouse and cellular models with pre-fibrotic state [11, 50]. As illustrated in Fig. 2C, the protein levels of COL-1, COL-3, and α-SMA were highest in the CD-NPs-induced cell model. In addition, cells in the CD-NPs-induced model had a spindle-shaped fibroblast-like morphology (Fig. 2E). These results indicate that the pre-fibrotic cell model was established. Further, the protein and mRNA levels of IGF1, p-IGF1R(Tyr1135/1136), and IGF1R were most activated in CD-NPs-induced pre-fibrosis model cells (Fig. 2C, D). In vivo, HE and Masson staining (Fig. 2F) showed more obvious nodules and blue collagen in the lung tissue of CD-NPs-induced model mice. The protein levels of COL-1, COL-3, and α-SMA were also highest in the lung tissue of CD-NPs-induced model mice (Fig. 2G). These results indicate that the pre-fibrotic mouse model was established. As in in vitro, IGF1, p-IGF1R(Tyr1135/1136), and IGF1R were highly activated in the lung tissue of CD-NPs-induced pre-fibrosis model mice (Fig. 2F, G). Taken together, the above results suggest that IGF1/IGF1R signaling is activated in both in vitro and in vivo pre-fibrosis models.

### Activation of IGF1/IGF1R signaling positively regulates CD-NPs-induced PF

**Fig. 3:**
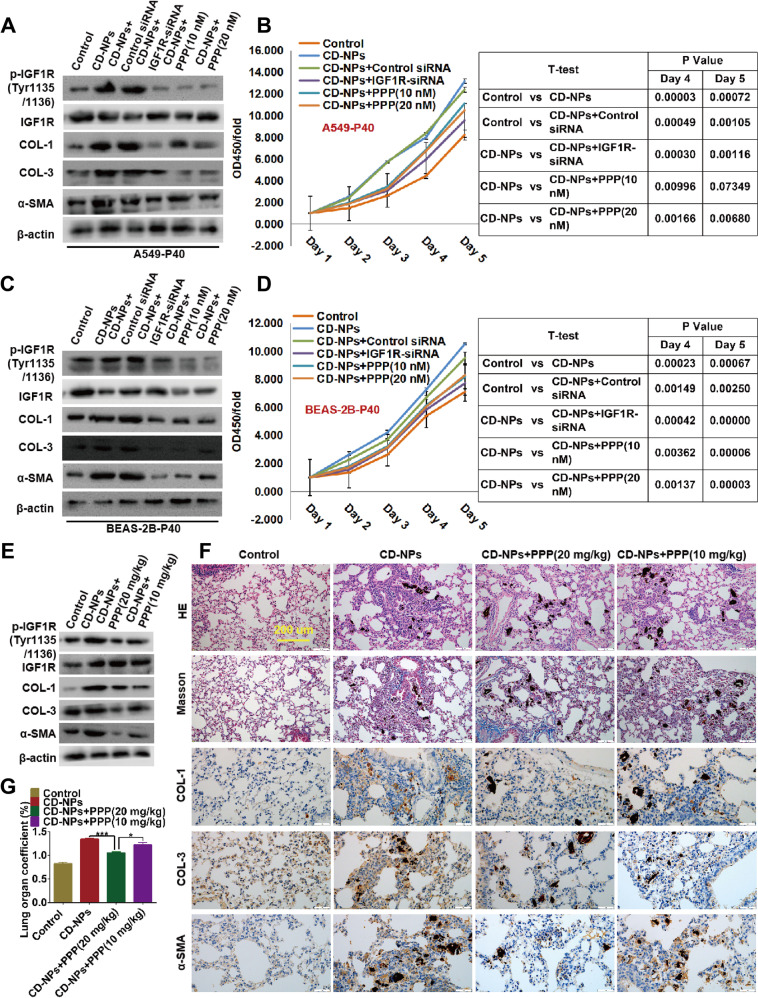
Activation of IGF1/IGF1R signaling positively regulates CD-NPs-induced PF. **A**, **C** After inhibition of IGF1R in an in vitro models of pre-fibrosis, IGF1/IGF1R signaling was detected by western blot. **B**, **D** After inhibition of IGF1R in an in vitro models of pre-fibrosis, cell viability was examined by CCK-8 assay. **E**, **F** IGF1/IGF1R signaling was detected by western blot and immunohistochemistry in an in vivo models of pre-fibrosis. **G** Lung coefficients in an in vivo pre-fibrotic model. Data were expressed as the mean ± SD, *n* = 3. *P < 0.05 and ***P < 0.001.

To explore the role of IGF1/IGF1R signaling in CD-NPs-induced PF, we performed intervention experiments using IGF1R-siRNA lentivirus and IGF1R inhibitor PPP. In vitro, as illustrated in Fig. 3A, C, compared with 40 passages of AECs not induced by CD-NPs (control group), the protein levels of p-IGF1R(Tyr1135/1136)/IGF1R, COL-1, COL-3, and α-SMA were significantly increased in CD-NPs-induced pre-fibrosis model cells (CD-NPs group). After silencing of IGF1R (CD-NPs+IGF1R-siRNA group) or treatment with PPP for 24 h (CD-NPs+PPP(10 nM) group and CD-NPs+PPP(20 nM) group) in CD-NPs-induced pre-fibrotic model cells, the levels of these proteins were significantly reduced and had dose-dependency of PPP. Figure 3B, D illustrate a similar trend in cell proliferation assays. In vivo, compared with the control group, the protein levels of p-IGF1R(Tyr1135/1136)/IGF1R, COL-1, COL-3, and α-SMA were increased in the CD-NPs group but decreased after PPP intervention; lung coefficients had a similar trend, and both had a dose-dependency of PPP (Fig. 3E–G). The results suggest that CD-NPs induce PF by activating IGF1/IGF1R signaling.

### Activation of IGF1/IGF1R signaling mediates EMT in in vitro and in vivo models by generation of ROS

**Fig. 4:**
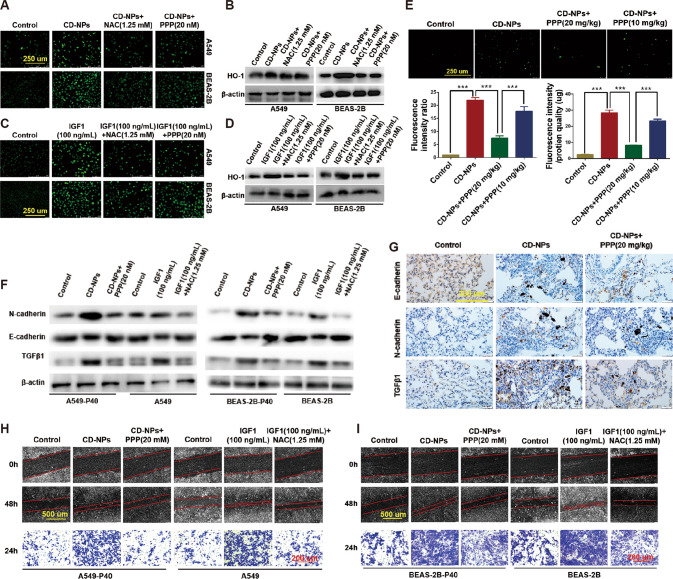
Activation of IGF1/IGF1R signaling mediates EMT in in vitro and in vivo models by promoting the generation of ROS. **A**, **C** ROS levels were detected by ROS fluorescent probe DCFH-DA in vitro. **B**, **D** The expression level of HO-1 was detected by western blot. **E** ROS levels were detected by ROS fluorescent probe DCFH-DA in vivo. Data were expressed as the mean ± SD, *n* = 3. ***P < 0.001. **F**, **G** The expression levels of EMT marker molecules (N-cadherin, E-cadherin and TGFβ1) was detected by western blot and immunohistochemistry. **H**, **I** The migration and invasion abilities of alveolar epithelial cells were detected by scratch healing assay and transwell assay.

Previous reports have shown that airborne particulate matter stimulated cells to induce ROS production [51, 52]. ROS can induce EMT, which is a key step in the pathogenesis of PF [53]. Therefore, we explored the relationship among IGF1/IGF1R signaling, ROS generation, and EMT in CD-NPs-induced PF. In vitro, as illustrated in Fig. 4A, compared with the control group, ROS were significantly increased in cells stimulated with 9.375 µg/mL of CD-NPs daily for 3 consecutive days (CD-NPs group), whereas ROS were decreased after three days of stimulation with the addition of the ROS inhibitor NAC (CD-NPs + NAC(1.25 mM) group) or the IGF1R inhibitor PPP (CD-NPs + PPP(20 nM) group) on the first day. Similarly, compared with the control group, ROS were significantly elevated in cells stimulated with recombinant human IGF1 for 24 h (IGF1(100 ng/mL) group), while ROS were decreased after 24 h of stimulation with NAC (IGF1;100 ng/mL) + NAC (1.25 mM) group or PPP (IGF1 100 ng/mL) + PPP (20 nM) group (Fig. 4C). The protein expression level of HO-1 can be upregulated after exposure to oxidative stress and cellular injury, which is strongly confirmed in Fig. 4B, D (the protein expression level of HO-1 follows the same trend as the intracellular ROS content). In vivo, compared with the control group, the production of ROS in lung tissue cells of CD-NPs-induced pre-fibrosis model mice was significantly increased, and ROS production decreased after the addition of PPP (Fig. 4E). These data suggest that IGF1/IGF1R signaling activation promotes CD-NPs-induced ROS production.

Furthermore, as illustrated in Fig. 4F, G, the EMT marker molecule N-cadherin and EMT driver TGFβ1 were significantly increased in CD-NPs-induced pre-fibrosis model cells and animal lung tissues, whereas the EMT marker molecule E-cadherin was decreased. After 24 h of PPP intervention, N-cadherin and TGFβ1 were decreased, and E-cadherin was increased. In addition, treatment of AECs with recombinant human IGF1 for 24 h induced increase of N-cadherin and TGFβ1 and decrease of E-cadherin, which could be reversed by NAC treatment for 24 h. Finally, the migration and invasion abilities of AECs were like the change in the expression levels of N-cadherin and TGFβ1 in each group (Fig. 4H, I). These results indicated that EMT occurred in pre-fibrosis model cells and animal lung tissue; recombinant human IGF1 could induce EMT in AECs; and inhibition of p-IGF1R/IGF1R levels and ROS generation could inhibit EMT.

Taken together, the above results suggest that activation of IGF1/IGF1R signaling mediates EMT in in vitro and in vivo models by promoting the generation of ROS.

### Inhibition of IGF1/IGF1R signaling alleviates CD-NPs-induced inflammation by inhibiting ROS-driven NF-κB/NLRP3 pathway

**Fig. 5:**
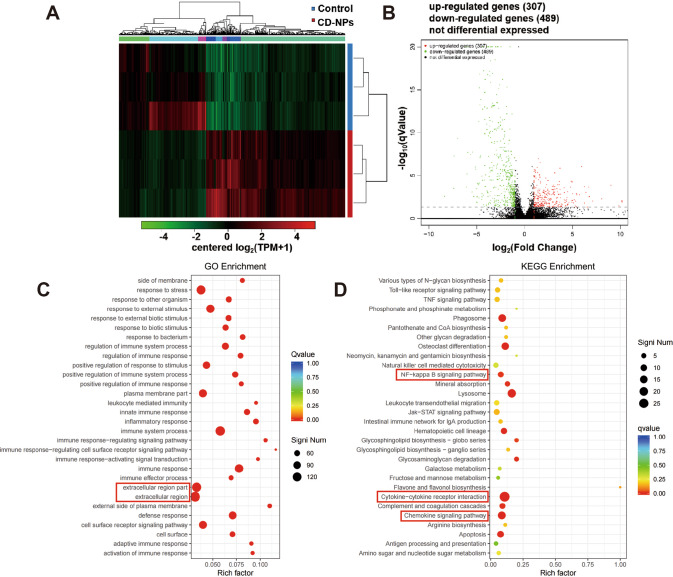
High-throughput gene sequencing analysis of lung tissue based on in vivo pre-fibrosis model mice. **A** Clustering heatmap of differential gene expression in normal mouse lung tissue and pre-fibrotic model mouse lung tissue. In the figure, each row represents a gene, and each column represents a sample. The color represents the expression level of the gene in the sample. Red represents the gene with high expression level in the sample, and green represents the low expression level. **B** Volcano plot of differential gene expression in normal mouse lung tissue and pre-fibrotic model mouse lung tissue. The horizontal axis is the fold-change value of the gene expression difference between samples in different groups, and the vertical axis is the statistically significant p Value representing the change in gene expression. The smaller the pValue, the larger the difference in −log_10_(pValue), the more obvious the difference. Each dot represents a gene, where red indicates upregulated genes, green indicates down-regulated genes, and black indicates non-differential genes. **C** GO enrichment analysis of differentially expressed genes. The vertical axis represents the functional annotation information, the horizontal axis represents the Rich factor corresponding to the function (the number of differential genes annotated to the function divided by the number of genes annotated to the function), and the red box marks the two functions with the most significant enrichment of differential genes. **D** KEGG-enrichment analysis of differentially expressed genes. The vertical axis represents the KEGG pathway information, the horizontal axis represents the Rich factor corresponding to the KEGG pathway (the number of differential genes annotated to the KEGG pathway divided by the number of genes annotated to the KEGG pathway), and the red boxes mark the three signaling pathways that are significantly enriched for the differential genes focused on in this study.

We next explored the possible changes in the signaling pathways involved in CD-NPs-induced PF. After selecting mouse lung tissue with obvious pre-fibrotic features, we analyzed DEGs and their GO and KEGG enrichment with high-throughput gene sequencing technology. As illustrated in Fig. 5A, there were significant differences in gene expression in the lung tissues of the control and CD-NPs group, whereas the gene expression trends were similar between the samples in each group. Compared with the control group, the CD-NPs group had 307 upregulated differential genes and 489 down-regulated differential genes (Fig. 5B). In addition, GO enrichment analysis revealed that differentially expressed genes were mainly enriched in the extracellular region (Fig. 5C). KEGG-enrichment analysis revealed that differentially expressed genes were mainly enriched in the NF-κB signaling pathway, the chemokine signaling pathway, and through cytokine-cytokine receptor interaction (Fig. 5D).

**Fig. 6:**
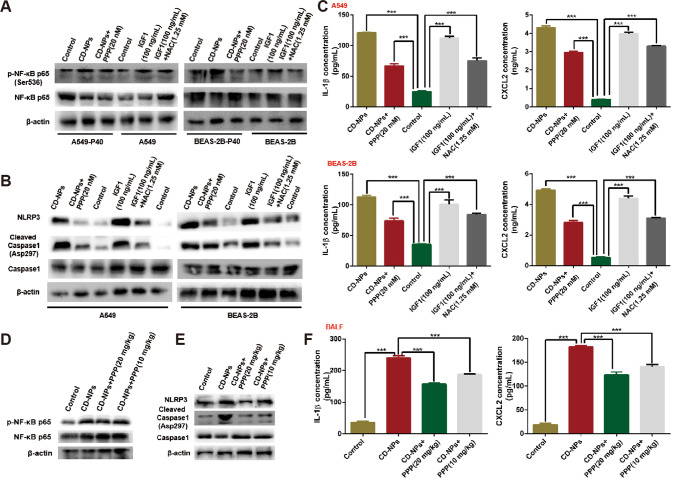
Inhibition of IGF1/IGF1R signaling can alleviate CD-NPs-induced inflammation by inhibiting ROS-driven NF-κB/NLRP3 pathway. **A**, **B** The activation level of NF-κB/NLRP3 pathway was detected by western blot in vitro. **C** Inflammatory factor IL-1β and inflammatory chemokine CXCL2 in the supernatant of alveolar epithelial cells were detected by ELISA. Data were expressed as the mean ± SD, *n* = 3. ***P < 0.001. **D**, **E** The activation level of NF-κB/NLRP3 pathway was detected by western blot in vivo. **F** Inflammatory factor IL-1β and inflammatory chemokine CXCL2 in mouse bronchoalveolar lavage fluid (BALF) were detected by ELISA. Data were expressed as the mean ± SD, *n* = 3. ***P < 0.001.

Previous studies have shown that intracellular ROS triggered by foreign substances can activate NF-κB signaling, resulting in transcriptional upregulation of inflammasome-related components (NLRP3, pro-IL-1β, and pro-IL-18), which in turn activate caspase-1 [31, 32]. Therefore, we explored whether inhibition of IGF1/IGF1R signaling could alleviate CD-NPs-induced inflammation by inhibiting the ROS-driven NF-κB/NLRP3 pathway. As illustrated in Fig. 6A, p-NF-κB(Ser536)/NF-κB was significantly increased in the CD-NPs-induced pre-fibrotic cell model and could be inhibited by PPP for 24 h; this result is evidence that inhibition of IGF1/IGF1R signaling to inhibit CD-NPs-induced PF could be achieved through NF-κB signaling. p-NF-κB(Ser536)/NF-κB was increased after 24 h stimulation of alveolar epithelial cells by recombinant human IGF1 and could be inhibited by NAC, indicating that ROS can activate NF-κB signaling (Fig. 6A). In addition, the protein levels of NLRP3 and Cleaved Caspase1(Asp297)/Caspase1 and the contents of IL-1β and CXCL2 in the supernatant were significantly increased in AECs stimulated with 9.375 µg/mL of CD-NPs daily for 3 consecutive days and with recombinant human IGF1 for 24 h, whereas these factors were suppressed by PPP and NAC (Fig. 6B, C). In vivo, the protein levels of p-NF-κB(Ser536)/NF-κB, NLRP3 and Cleaved Caspase1(Asp297)/Caspase1 in the lung tissues, as well as the contents of IL-1β and CXCL2 in the bronchoalveolar lavage fluid, were significantly increased in CD-NPs-induced pre-fibrosis model mice and inhibited by concentration-dependent PPP (Fig. 6D–F). These results support the hypothesis that inhibition of IGF1/IGF1R signaling alleviate CD-NPs-induced inflammation by inhibiting the ROS-driven NF-κB/NLRP3 pathway.

### Inhibition of NLRP3 reverses the effects of CD-NPs on inflammation and EMT

**Fig. 7:**
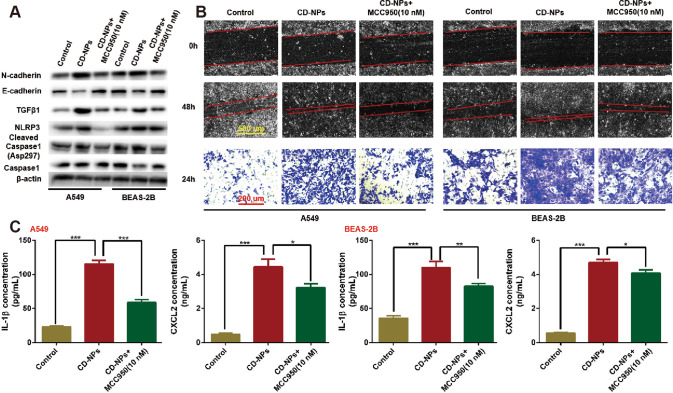
Inhibition of NLRP3 reverses the effects of CD-NPs on inflammation and EMT in vitro. **A** The expression levels of EMT marker molecules (N-cadherin, E-cadherin and TGFβ1) and inflammatory pathway protein molecules (NLRP3, Cleaved Caspase1/Caspase1) were detected by western blot. **B** The migration and invasion abilities of alveolar epithelial cells were detected by scratch healing assay and transwell assay. **C** Inflammatory factor IL-1β and inflammatory chemokine CXCL2 the supernatant of alveolar epithelial cells were detected by ELISA. Data were expressed as the mean ± SD, *n* = 3. *P < 0.05, **P < 0.01 and ***P < 0.001.

To test the role of NLRP3 in CD-NPs-induced inflammation and EMT, we added the NLRP3 inhibitor MCC950 on the first day for three days to cells stimulated with 9.375 μg/mL of CD-NPs daily for 3 consecutive days. As illustrated in Fig. 7A, compared with the control group, the epithelial marker molecule E-cadherin was decreased in the CD-NPs group, and the mesenchymal marker molecule N-cadherin and the EMT driver TGFβ1 were increased, and MCC950 reversed this phenomenon. In addition, the migration and invasion abilities of AECs in each group had similar trends with mesenchymal marker molecules and EMT drivers after treatment (Fig. 7B). The above data indicated that inhibition of NLRP3 reversed EMT induced by CD-NPs. We also observed that, compared with the normal group, the protein levels of NLRP3 and Cleaved Caspase1(Asp297)/Caspase1 (Fig. 7A) and the contents of IL-1β and CXCL2 in the supernatant (Fig. 7C) were significantly increased in the CD-NPs group and were reduced by MCC950, a result indicating that inhibition of NLRP3 reversed the inflammation induced by CD-NPs.

### ROS-mediated AKT/GSK3β signals drive NF-κB/NLRP3 pathway to promote inflammation and EMT

**Fig. 8:**
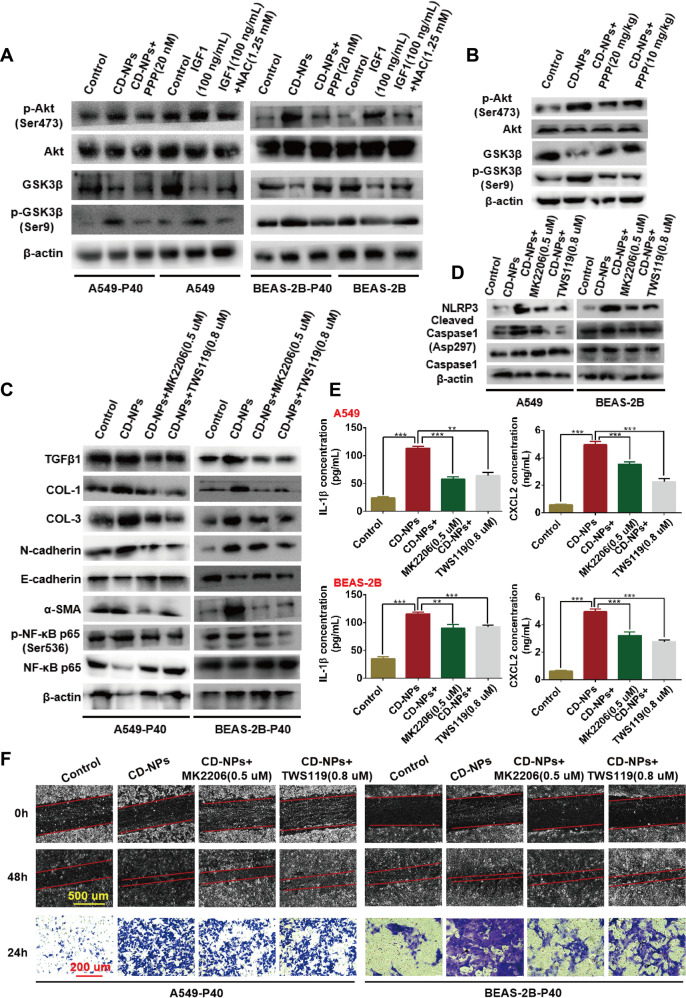
ROS-mediated AKT/GSK3β signals drive NF-κB/NLRP3 pathway to promote inflammation and EMT. **A**, **B** The activation level of AKT/GSK3β pathway was detected by western blot in vitro and in vivo. **C**, **D** The expression levels of EMT marker molecules (N-cadherin, E-cadherin and TGFβ1), inflammatory pathway protein molecules (NLRP3, Cleaved Caspase1/Caspase1) and fibrosis marker molecules (COL-1, COL-3 and α-SMA) was detected by western blot. **E** Inflammatory factor IL-1β and inflammatory chemokine CXCL2 the supernatant of alveolar epithelial cells were detected by ELISA. Data were expressed as the mean ± SD, *n* = 3. **P < 0.01 and ***P < 0.001. **F** The migration and invasion abilities of alveolar epithelial cells were detected by scratch healing assay and transwell assay.

Finally, we explored whether AKT/GSK3β signaling has a mediating role between CD-NPs-induced ROS generation and the NF-κB/NLRP3 pathway. As illustrated in Fig. 8A (Add PPP to CD-NPs-induced passage 40 cells for 24 h) and B, the amounts of p-Akt(Ser473)/Akt, p-GSK3β(Ser9)/GSK3β were increased in the CD-NPs-induced pre-fibrosis model in vitro and in vivo compared with amounts in the control group; this effect was inhibited by PPP, indicating that CD-NPs can induce IGF1/IGF1R signaling-mediated PF through AKT/GSK3β signaling. Furthermore, recombinant human IGF1-stimulated AKT/GSK3β signals could be inhibited by NAC for 24 h, indicating that ROS induced by IGF1/IGF1R signaling could activate AKT/GSK3β signal (Fig. 8A). We added MK2206 and TWS119 to CD-NPs-induced passage 40 cells for 24 h. Then, inhibition of AKT and GSK3β phosphorylation by MK2206 and TWS119 inhibited CD-NPs-induced EMT (Fig. 8C, F) and collagen formation (Fig. 8C) via the NF-κB/NLRP3 pathway. As illustrated in Fig. 8D, E, we added MK2206 and TWS119 on the first day for three days to cells stimulated with 9.375 μg/mL of CD-NPs daily for 3 consecutive days. Then, inhibition of AKT and GSK3β phosphorylation by MK2206 and TWS119 inhibited CD-NPs-induced inflammation (Fig. 8D, E) via the NF-κB/NLRP3 pathway.

## Discussion

The biggest hazard of coal mining processes is the generation of CD that can harm the health of coal miners [3, 5]. Although the incidence of reduced by measures such as lowering CD exposure limits in countries around the world has rebounded in recent years [6], few studies have focused on the impact of CD-NPs on coal workers’ pneumoconiosis. In this study, (1) IGF1/IGF1R signaling plays an important role in CD-NPs-induced PF; (2) Activation of IGF1/IGF1R signaling promotes CD-NPs-induced ROS production; (3) ROS production promotes CD-NPs through the NF-κB/NLRP3 pathway NPs-induced inflammation and EMT; and (4) AKT/GSK3β signal mediates ROS and the NF-κB/NLRP3 pathway.

When the particle size is more than 1 µm, most particles are deposited in the upper respiratory tract due to inertial mechanisms. As particle size decreases, particle deposition is controlled by the competition of inertial and Brownian diffusion, resulting in small particles that penetrate deep into the lungs, which in turn have higher pulmonary toxicity than do larger particles [54, 55]. Also, small particles have larger surface area. The large surface area of nanoparticles endows them with greater biological activity, possibly inducing more pronounced oxidative stress or cellular dysfunction [54, 56]. The PDI in particle size distribution refers to the uniformity of particle size distribution. When the PDI value is less than 0.1, it can be considered monodisperse particles; that is, the particle size is relatively uniform; when the PDI value exceeds 0.7, the particle size distribution can be considered very broad [57, 58]. In this study, the PDI values of CD-NPs (PDI = 0.343) and CD-MPs (PDI = 0.333) were both greater than 0.1 but less than 0.7, and there was no significant difference between them. Combined with the analysis of particle size distribution, both CD-NPs (<1 µm) and CD-MPs (1 µm < CD-MPs <5 µm) are medium-dispersed substances with relatively uneven particle size distribution. In additon, the Zeta potentials of CD-NPs and CD-MPs may also lead to non-uniform particle size of the CD, which may agglomerate in the lung fluid and affect cellular uptake. However, since the in vitro detection system and the in vivo absorption and metabolism system are two separate environments, whether CD accumulates in the lung fluid and affects cellular uptake has not been determined. When poorly soluble particles remain in the lungs, they can cause oxidative stress, leading to inflammation, fibrosis, or cancer [59, 60]. Thus, CD-NPs are more pulmonary toxic than CD-MPs, which was also confirmed in our previous research [11]. However, the mechanism by which CD-NPs induce PF remains unclear.

The GEO database is a gene expression database created by the National Center for Biotechnology Information in 2000. It contains gene expression data, mainly gene chips and high-throughput sequencing data submitted by research institutions around the world. The data on gene expression detection in the published papers can be found through the GEO database. In addition, the occurrence of diseases must involve changes in genes. Therefore, we first extensively and rigorously screened 5 PF datasets from the GEO database and performed a screen for intersecting differential genes. Combining eight computational methods, the upregulated genes IGF1, POSTN, MMP7, ASPN and CXCL14 were initially selected as hub genes; those with the highest frequency and score of IGF1, POSTN, and MMP7 were selected as hub genes. In support of this selection, IGF1 [45–47], POSTN [61], MMP7 [62] have been reported to promote PF.

PF is characterized by damage to AECs, excessive deposition of ECM, and accumulation of myofibroblasts. Myofibroblasts are the main cells in fibrotic lesions. They are transformed by fibroblast proliferation and are usually spindle-shaped or star-shaped. During the formation of PF, the differentiation of lung fibroblasts into myofibroblasts cooperates with the upregulation of α-SMA expression and the synthesis of collagens to form the ECM [12]. Another characteristic of PF is increased organ coefficient. CD-induced PF has a large lung organ coefficient [63]. Therefore, in this study, we examined the levels of α-SMA, COL-1 and COL-3, cell morphology, cell proliferation ability, nodule and collagen content, and lung organ coefficient in lung tissue to learn whether the pre-fibrosis model had been constructed in vitro and in vivo and the role of IGF1/IGF1R signaling activation in PF.

Intermittent entry of foreign particles into the lung can cause repetitive damage to the AECs. In the present study, we demonstrated that EMT, which indicates abnormal damage repair, is present in CD-NPs-induced PF and is regulated by IGF1/IGF1R and its induced ROS. High-throughput gene sequencing and its bioinformatics analysis technology are important techniques for study of the changes of disease genes and gene functions as well as changes in cell signaling pathways. In this study, high-throughput gene sequencing and bioinformatics analysis of mouse lung tissue revealed that cytokine-cytokine receptor interaction, NF-κB signaling pathway and chemokine signaling pathway are involved in CD-NPs-induced PF. IGF1/IGF1R signaling activation validates cytokine-cytokine receptor interaction. The NF-κB signaling pathway is closely related to ROS, and ROS can promote inflammation through the NF-κB/NLRP3 pathway [31]. Other reports suggest that inflammation has a positive driving effect during EMT [64, 65]. In the present study, we confirmed that IGF1/IGF1R-induced ROS could promote inflammation and EMT through the NF-κB/NLRP3 pathway, thereby promoting CD-NPs-induced PF. Foreign substances can activate the NF-κB/NLRP3 pathway and promote inflammation by targeting Toll-like receptors [66, 67]. High-throughput gene sequencing and bioinformatics analysis of mouse lung tissue in this study also showed that the Toll-like receptor signaling pathway was activated by CD-NPs, but there was no significant difference (Pvalue=0.03; Qvalue=0.28). Consistent with previous reports [33], the inflammatory chemokine CXCL2 is recruited upon activation of the NLRP3 inflammasome.

Myofibroblasts can secrete TGFβ1, monocyte chemokine and other cytokines, which are also inflammatory mediators. In the process of PF, these cytokines secreted by myofibroblasts form a positive feedback inflammatory response by promoting the aggregation of inflammatory cells and enhancing inflammation. The high-level expression of fibrogenic factors such as TGFβ1 promotes the persistence of these inflammatory responses and increases the damage of AECs throughout the entire course of fibrosis. Given that TGFβ1 has been shown to induce EMT in PF [26–28], we only used it as a marker molecule to detect EMT and did not explore its relationship with inflammation. Inflammation and EMT may be a positive feedback loop in PF, and it is necessary to find a common upstream switch for both, which may be IGF1/IGF1R/ROS. Furthermore, EMT confers the ability of cells to metastasize and invade, and it is involved in tissue healing and organ fibrosis [21, 68]. In this study, in addition to the EMT marker molecules E-cadherin and N-cadherin, we detected the cell migration and invasion ability that characterizes EMT.

Yang et al. [41] proposed that activation of ROS/AKT signaling promotes inflammation and fibrosis in the lung. It was previously reported that phosphorylation of Ser/Thr protein kinase at the Ser9 site inhibited GSK3β activation and suggested that NF-κB signaling can be activated by AKT activation and GSK3β inactivation [42, 43]. Therefore, we explored the mediating role of AKT/GSK3β between ROS and NF-κB/NLRP3 signaling. We found that ROS could promote AKT activation through phosphorylation at Ser473. Consistent with previous reports [42, 43], activated AKT in turn inhibited GSK3β activation through phosphorylation at Ser9. In addition, ROS promoted the activation of NF-κB/NLRP3 signaling and its activation-mediated inflammation and EMT by activating AKT and p-GSK3β(Ser9), thereby promoting the induction of PF by CD-NPs. The above results indicated that AKT/GSK3β mediates the relationship between ROS and NF-κB/NLRP3 signaling.

CD-NPs could directly contact and injure the bronchus or alveolar epithelium after inhalation, and repeated damage to lung epithelium is considered one of the inducers of PF [20]. Many data show that AEC II are multifunctional cells with stem cell potential, self-renewal, and participation in PF. A549 is a human lung adenocarcinoma epithelial cell that can synthesize lecithin, contains high unsaturated fatty acids, plays an important role in maintaining cell membrane phospholipids, and has the characteristics of AEC II. Therefore, A549 is widely used as an in vitro model of AEC II [69–71]. BEAS-2B is a bronchial epithelial cell line that can promote PF progression by EMT [72]. Therefore, BEAS-2B and A549 cells were used in this study to investigate the relationship between CD-NPs-treated lung epithelial cells and PF. Notably, since A549 is derived from cancer tissue and BEAS-2B is a normal bronchial epithelial cell, these cell lines have slight differences in protein expression. For example, the expression levels of COL-1, COL-3, α-SMA and N-cadherin in A549 cells were slightly higher than those in BEAS-2B cells. Lipid peroxidation, GSH depletion, and SLC7A11 inhibition are common causes of ferroptosis in A549 cells [73]. Studies have found that attenuation of ferroptosis contributes to the progression of PF [74]. Therefore, genetic modifications, such as anti-ferroptosis in A549 cells, may have contributed to this slight difference.

This study has limitations: We only investigated the mechanism of CD-NPs-induced PF and did not deeply compare the differences in the mechanism of CD-NPs and CD-MPs in the induction of PF. In addition, only through high-throughput gene sequencing and bioinformatics analysis of mouse lung tissue was CD-NPs activation of the Toll-like receptor signaling pathway found, and the difference was not statistically significant, so we did not attempt in vivo or in vitro verification. Although we found that CD-NPs induce PF more strongly than CD-MPs and explored the possible molecular mechanism, details of its molecular mechanism needs further analysis.

## Conclusions

This study, based on in vitro and in vivo models of pre-coal dust fibrosis, demonstrated for the first time that coal dust nanoparticles induced pulmonary fibrosis by promoting inflammation and epithelial-mesenchymal transition via the NF-κB/NLRP3 pathway driven by IGF1/ROS-mediated AKT/GSK3β signals. These findings may provide a valuable experimental reference for the prevention and treatment of coal workers’ pneumoconiosis.

## Materials and methods

### Cell lines and reagents

Sources and cultures of human normal bronchial epithelial BEAS-2B cells and alveolar type II epithelial A549 cells have been described in detail in our previous research [11]. IGF1R was silenced in 40 passages of AECs induced by CD-NPs (IGF1R-siRNA) with use of knockout lentiviruses according to the manufacturer’s instructions (Sangon Biotechnology Co., Ltd., Shanghai, China).

Picropodophyllin (PPP, AXL1717) (a selective IGF-1R inhibitor), MK2206 (a specific inhibitor of AKT) and TWS119 (a specific inhibitor of GSK3β) were procured from Selleck Chemicals (Houston, USA). Recombinant human IGF1 was obtained from PeproTech (New Jersey, USA). N-Acetylcysteine (NAC) (a ROS inhibitor) and MCC950 (a potent and selective NLRP3 inhibitor) were bought from MedChemExpress (New Jersey, USA).

### Nano-to-micron sized respirable coal dust collection and preparation

The collection and preparation of CD-NPs and CD-MPs as well as the analytical methods for their particle size distribution, Zeta potential and elemental composition have been described in detail in our previous research [11]. The polydispersity index (PDI) is an important data in the results of CD particle size distribution analysis.

### Screening hub genes

Using “pulmonary fibrosis” as the key word, we selected the target dataset via the GEO2R online analysis tool from the GEO database through three rounds of four persons. Then, adjusted *P* < 0.05 and |logFC| ≥ 1.0 were used as the criteria for selection of the differential genes (DEGs) of each dataset, and the jvenn online tool was taken to determine the intersection DEGs. Protein-protein interaction (PPI) network analysis of the intersection DEGs was performed with STRING and Cytoscape software. The hub genes were predicted by the plug-in cytoHubba of Cytoscape software. The parameters of PPI analysis were Network type, full STRING network; meaning of network edges, evidence; active interaction evidence, all; minimum required interaction score, low confidence (0.150). The parameters of cytoHubba were the top three nodes ranked by MCC, DMNC, MNC, Degree, EPC, BottleNeck, EcCentricity, and Closeness; display options, all.

### Animals

The origin and husbandry conditions of C57BL/6 male mice and the ethics of animal experimentation have been described in detail in our previous research [11]. Fifteen mice were randomly divided into 5 groups: The control group was injected intranasally with 40 μL phosphate buffered saline (PBS) per day; the CD-NPs group was injected intranasally with 32 μg CD-NPs suspension per day; the CD-MPs group was injected intranasally with 32 μg CD-MPs suspension per day; the CD-NPs + PPP (20 mg/kg) group was injected intranasally with 32 μg CD-NPs suspension combined with 100 uL PPP per day; and the CD-NPs+PPP (10 mg/kg) group were injected intranasally with 32 μg CD-NPs suspension combined with 100 uL PPP per day for 12 weeks. Finally, the animals were dissected, and lung organ coefficient was calculated: lung organ coefficient = lung tissue mass (g) / animal body weight (g) × 100%. Lung tissue was collected for hematoxylin-eosin (HE) staining, MASSON staining and immunohistochemistry (IHC) provided by Hefei Xinle Biotechnology Co., Ltd.). Bronchoalveolar lavage fluid was collected for measuring IL-1β and CXCL2 levels by using ELISA kits, as described in our previous research [11].

### Western blot

The specific procedures for cell and tissue protein extraction, quantification and western blot have been described in detail in our previous research [11]. The information of antibodies is as follows: IGF1 (1: 1000, Abclonal, #A11985, China), IGF1R (1: 1000, CST, #9750, USA), p-IGF1R(Tyr1135/1136) (1: 1000, CST, #3024, USA), COL-1 (1: 2000, Abcam, ab260043, UK), COL-3 (1: 1000, Abcam, ab184993, UK), α-SMA (1:1000, CST, #19425, USA), heme oxygenase-1 (HO-1, 1: 1000, CST, #5853, USA), N-cadherin (1: 1000, CST, #49398, USA), E-cadherin (1: 1000, CST, #49398, USA), TGFβ1 (1: 1000, abclonal, #A2124, China), p-NF-kB p65(Ser536) (1: 1000, CST, #3033, USA), NF-kB p65 (1: 1000, CST, #8242, USA), NLRP3 (1: 1000, CST, #15101, USA), Cleaved Caspase1(Asp297) (1: 1000, CST, #4199, USA), Caspase1 (1: 1000, CST, #2225, USA), p-Akt(Ser473) (1: 1000, CST, #4060, USA), Akt (1: 1000, CST, #4691, USA), p-GSK3β(Ser9) (1: 1000, CST, #9322, USA), GSK3β (1: 1000, CST, #9369, USA) and β-actin (1: 1000, CST, #4970, USA).

### Quantitative real-time PCR (qRT-PCR)

Cellular RNA was extracted and measured. According to the instructions of PrimeScript™ RT Master Mix (#RR036A, TaKaRa, Japan), a reverse transcription reaction system was prepared to obtain cDNA, and an amplification system was prepared for real-time detection of qPCR gene expression. Relative gene expression levels were presented as the 2^−ΔΔCt^ and normalized GAPDH as internal controls. The primer sequences of IGF1 used for qRT-PCR were Forward: 5′-TGTACTGTGCGCCTGCCAAGACTA-3′; Reverse: 5′-TGCTTGTGCTGTCCTACGCTCTGT-3′. The primer sequences of IGF1R used for qRT-PCR were: Forward: 5′-TCGACATCCGCAACGACTATC-3′; Reverse: 5′-CCAGGGCGTAGTTGTAGAAGAG-3′. The primer sequences of GAPDH used for qRT-PCR were: Forward: 5′-CTGGGCTACACTGAGCACC-3′; Reverse: 5′-AAGTGGTCGTTGAGGGCAATG-3′.

### CCK-8 assay, cell migration and invasion assays and ELISA assay in vitro

In the CD-NPs-induced pre-fibrotic model cells, we tested the proliferative capacity of the cells with the CCK-8 assay after inhibition of IGF1R by PPP treatment for 24 h or silencing of IGF1R with lentivirus. In addition, the specific procedures for CCK-8 assays, cell migration and invasion assays, and in vitro ELISA assays have been described in detail in our previous research [11]

### Determination of ROS level

In vitro, cells were treated with 9.375 µg/mL of CD-NPs daily for 3 days and treated with 1.25 mM NAC or 20 nM PPP on the first day. Additionally, cells were treated with 100 ng/mL recombinant human IGF1 for 24 h, followed by co-treatment with 1.25 mM NAC or 20 nM PPP for 24 h. The ROS fluorescent probe working solution was added to a 24-well plate and incubated for 30 min in the dark. Finally, images were acquired under an inverted fluorescence microscope.

In vivo, the selected lung tissue was immediately washed twice with pre-cooled PBS. Necrotic components, fibers, fat, and blood vessels were removed from the tissue mass. Tissue blocks were cut into small pieces of about 1 mm^3^ with ophthalmic scissors and rinsed with pre-cooled PBS. Trypsin was added, and the cells were incubated at 37 °C for 30 min. The digestion was terminated with pre-cooled PBS, and the tissue blocks were removed by filtration through a 300-mesh nylon mesh. The filtered cells were collected and single-cell suspensions were prepared by resuspending in PBS, and the cell count in each group was adjusted to 1.213 × 10^6^.

### High-throughput gene sequencing analysis

Lung tissue was collected for high-throughput gene sequencing analysis provided by Sangon Bioengineering Co., Ltd. (Shanghai, China). DEGs were selected according to adjusted *P* < 0.05 and |logFC| ≥ 1.0, and then used for differential gene expression cluster analysis, KEGG, and GO enrichment analysis.

### Statistical analysis

Three independent replicate trials were conducted in this study. Data are presented as mean ± SD. Statistical analysis was performed using Student’s *t* test (two groups) or one-way analysis of variance (multiple groups). *P* < 0.05 were considered statistically significant.

## Supplementary information

Supplementary figures and legends

Original Data File

## Data Availability

All data are available in the main text or the supplementary materials.
