# Supplementary material for: Coal dust nanoparticles induced pulmonary fibrosis by promoting inflammation and epithelial-mesenchymal transition via the NF-κB/NLRP3 pathway driven by IGF1/ROS-mediated AKT/GSK3β signals
Source: Cell Death Discov. 2022 Dec 29;8:500. doi: 10.1038/s41420-022-01291-z (PMC9800584; doi:10.1038/s41420-022-01291-z)
Supplement: Supplementary file 1 — Supplementary figures and legends [file 41420_2022_1291_MOESM1_ESM.pdf]

Coal dust nanoparticles induced pulmonary fibrosis by promoting inflammation and epithelial-mesenchymal transition via the NF-κB/NLRP3 pathway driven by IGF1/ROS-mediated AKT/GSK3β signals

CD-NPs			Mean (mV)	Area (%)	St Dev (mV)
Zeta Potential (mV):	20.9	Peak 1:	20.1	96.0	6.26
Zeta Deviation (mV):	7.50	Peak 2:	42.3	4.0	3.19
Conductivity (mS/cm):	0.535	Peak 3:	0.00	0.0	0.00

Result quality : See result quality report

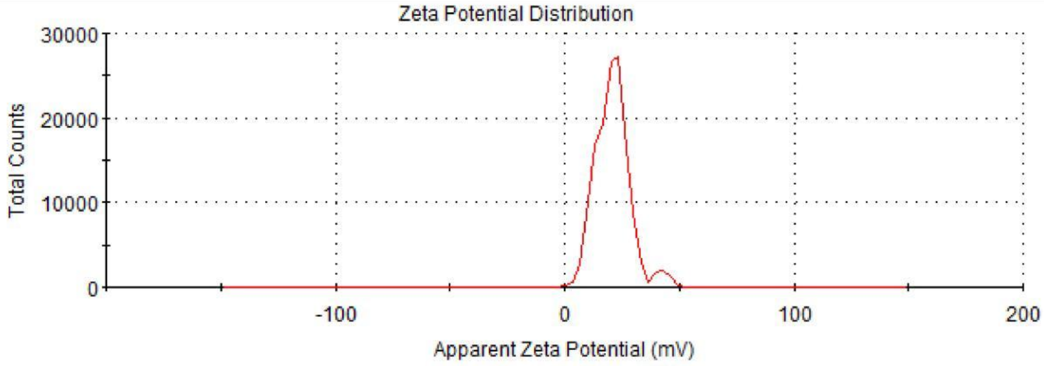

CD-MPs			Mean (mV)	Area (%)	St Dev (mV)
Zeta Potential (mV):	-18.6	Peak 1:	-20.6	89.9	7.01
Zeta Deviation (mV):	9.48	Peak 2:	2.03	10.1	4.12
Conductivity (mS/cm):	0.0385	Peak 3:	0.00	0.0	0.00

Result quality : See result quality report

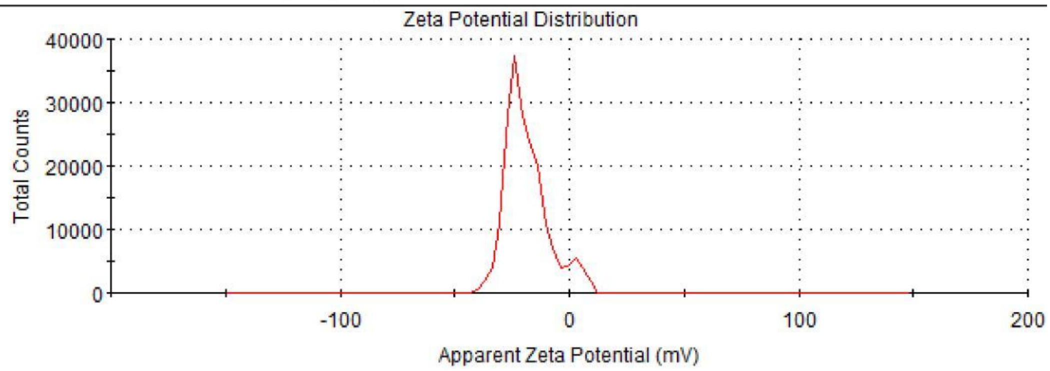

Figure S1 The Zeta potential of CD-MPs and CD-NPs were measured with a Malvern Nanoparticle Size Potentiometer.

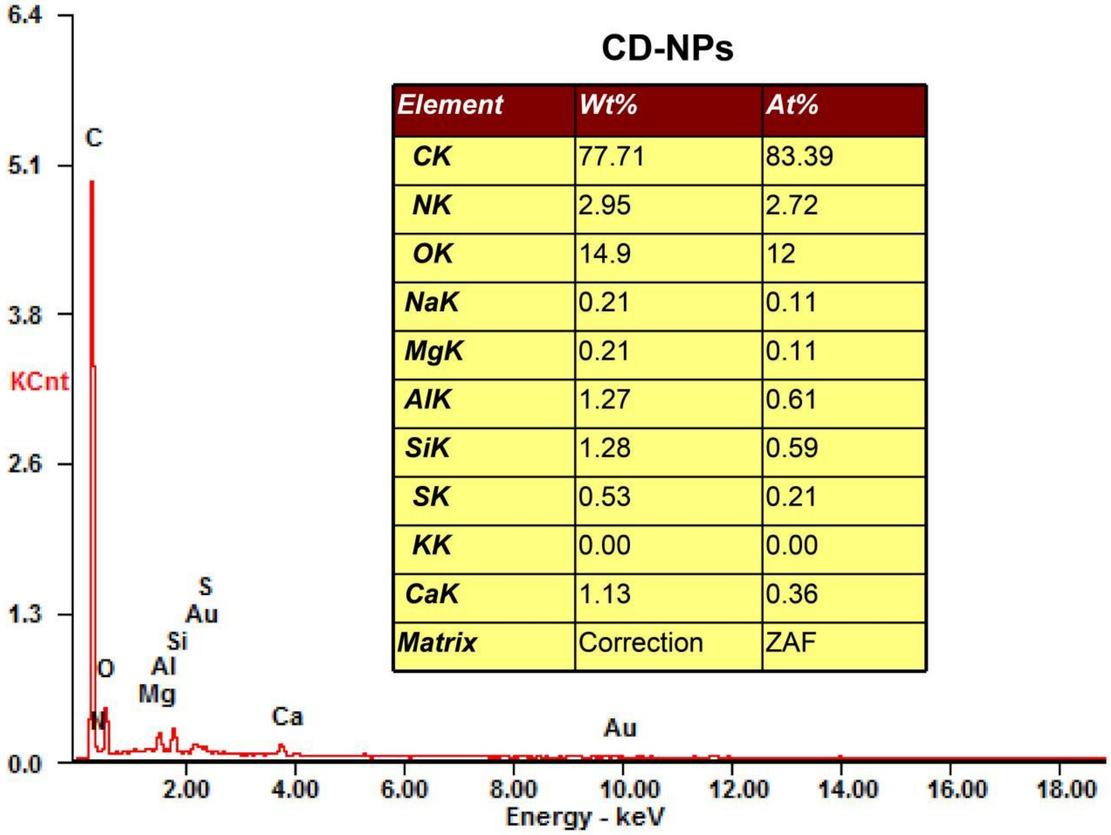

Element	Wt%	At%
CK	77.71	83.39
NK	2.95	2.72
OK	14.9	12
NaK	0.21	0.11
MgK	0.21	0.11
AlK	1.27	0.61
SiK	1.28	0.59
SK	0.53	0.21
KK	0.00	0.00
CaK	1.13	0.36
Matrix	Correction	ZAF

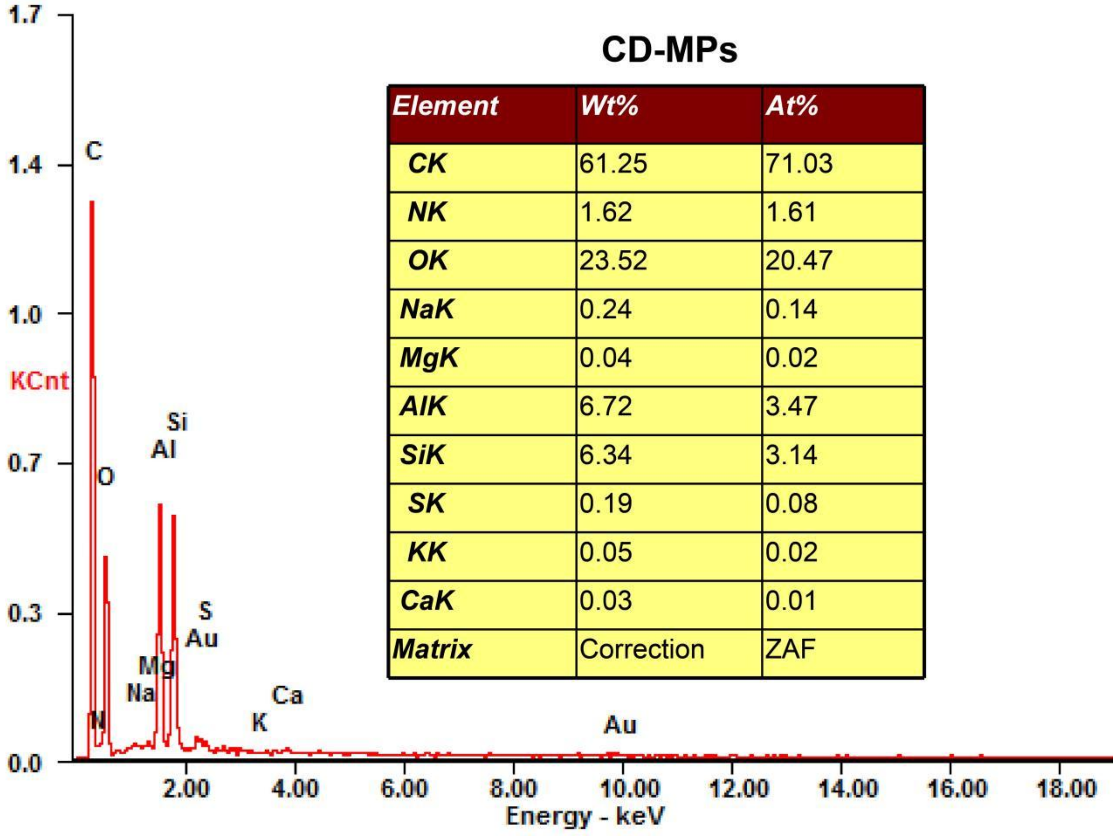

Element	Wt%	At%
CK	61.25	71.03
NK	1.62	1.61
OK	23.52	20.47
NaK	0.24	0.14
MgK	0.04	0.02
AlK	6.72	3.47
SiK	6.34	3.14
SK	0.19	0.08
KK	0.05	0.02
CaK	0.03	0.01
Matrix	Correction	ZAF

Figure S2 The elemental composition of CD-NPs was analyzed with energy X-ray spectrometer (EDAX-GENESIS, USA) at 20 keV with an analysis area of 1.56 μm^2.
