# Supplementary material for: Coal dust nanoparticles induced pulmonary fibrosis by promoting inflammation and epithelial-mesenchymal transition via the NF-κB/NLRP3 pathway driven by IGF1/ROS-mediated AKT/GSK3β signals
Source: Cell Death Discov. 2022 Dec 29;8:500. doi: 10.1038/s41420-022-01291-z (PMC9800584; doi:10.1038/s41420-022-01291-z)

Coal dust nanoparticles induced pulmonary fibrosis by promoting inflammation and EMT via the NF-κB/NLRP3 pathway driven by IGF1/ROS-mediated AKT/GSK3β signals

Fig 2C

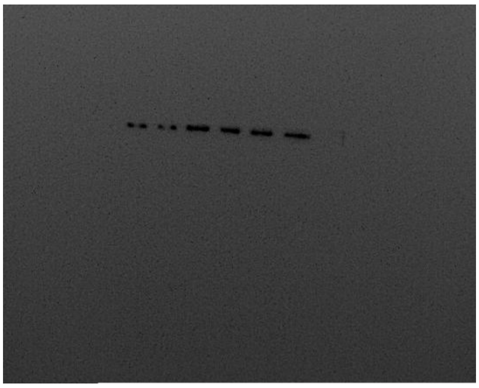

IGF1

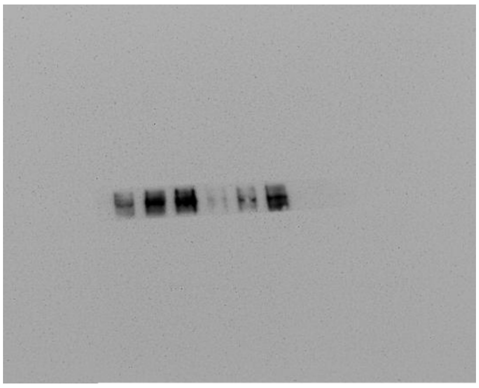

p-IGF1R(Thr1135/1136)

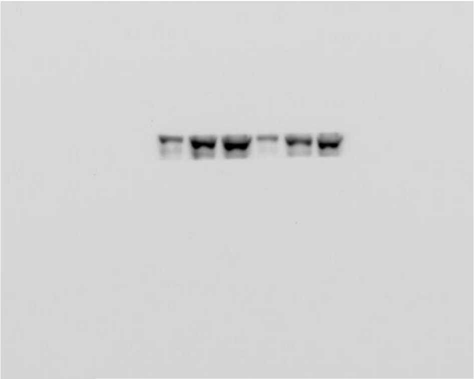

IGF1R

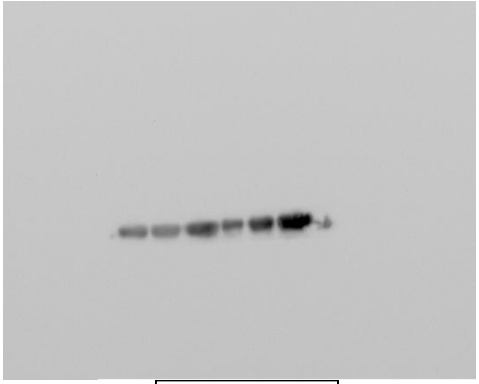

COL-1

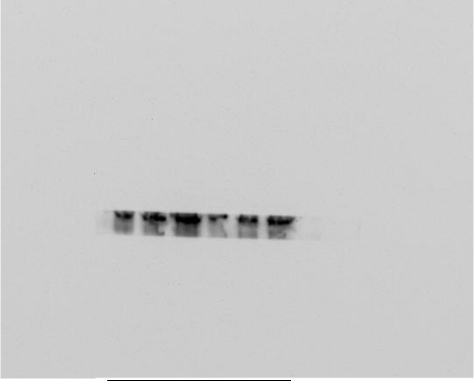

COL-3

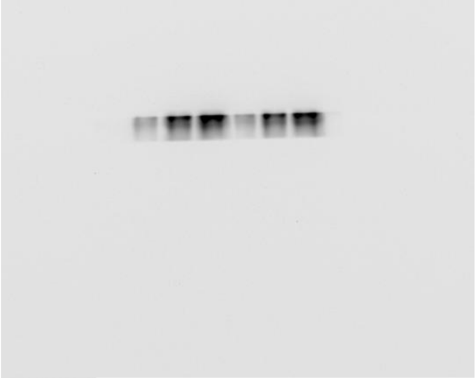

α-SMA

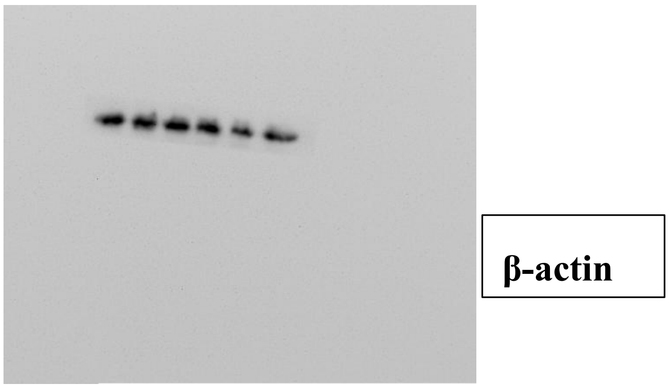

β-actin

Fig 2G

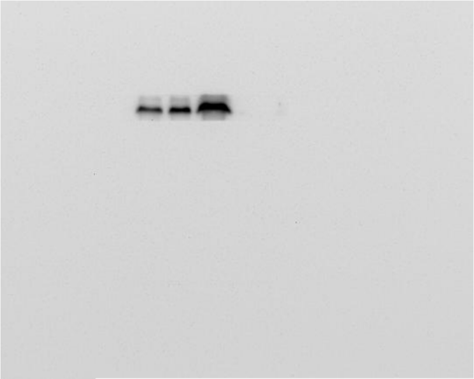

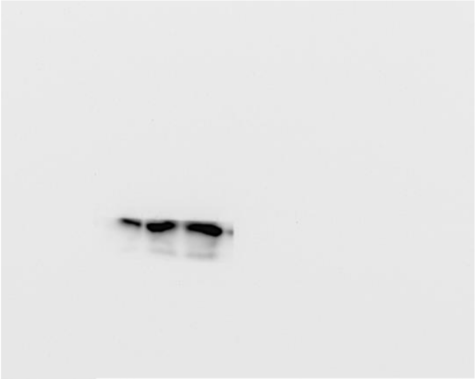

IGF1

p-IGF1R(Thr1135/1136)

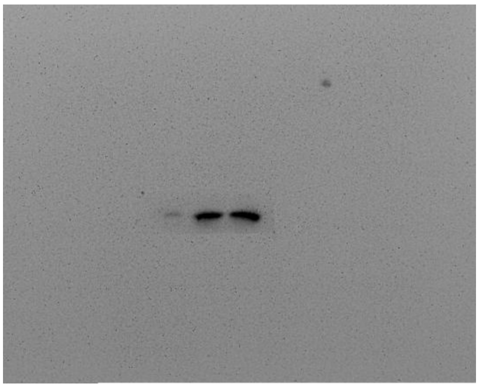

IGF1R

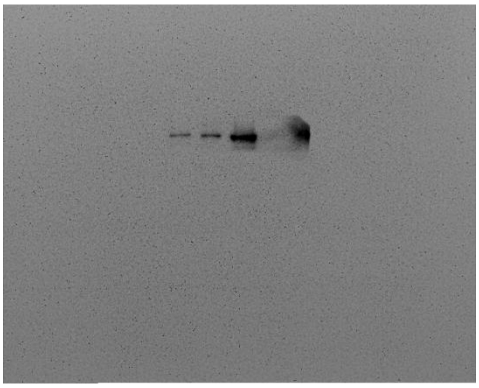

COL-1

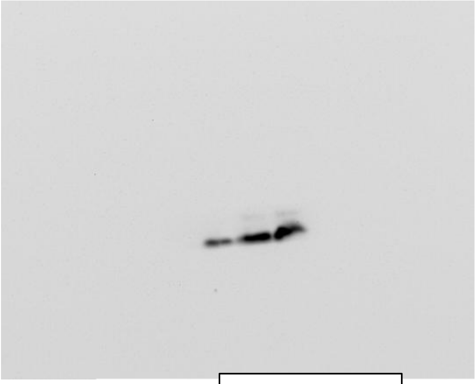

COL-3

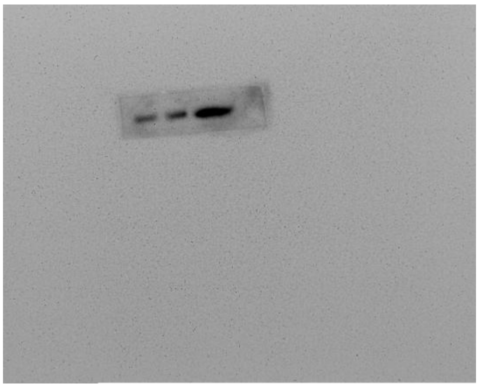

α-SMA

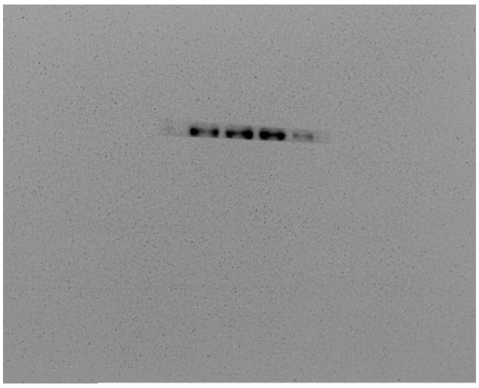

β-actin

Fig 3A

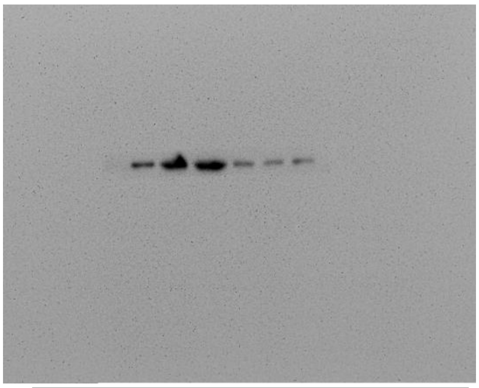

p-IGF1R(Thr1135/1136)

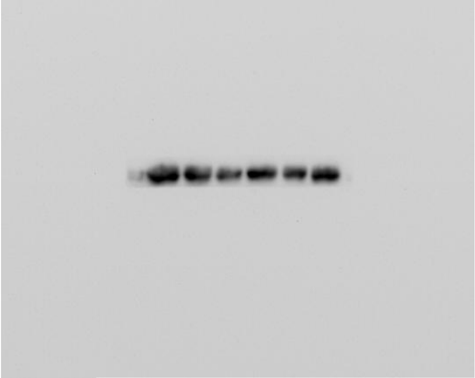

IGF1R

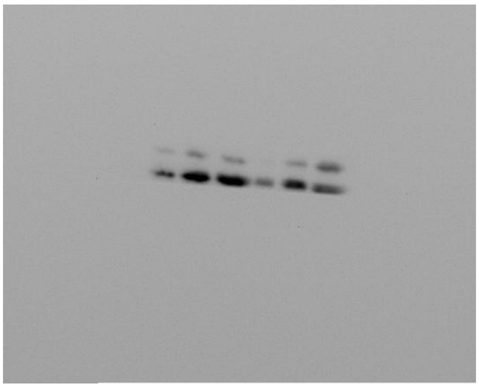

COL-1

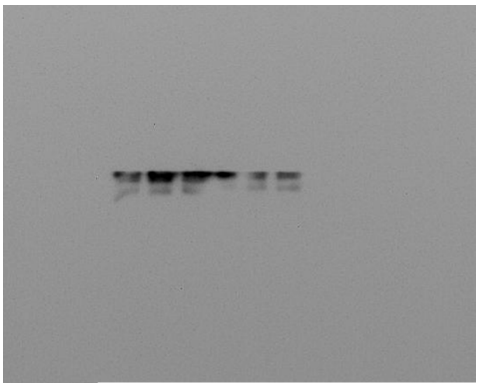

COL-3

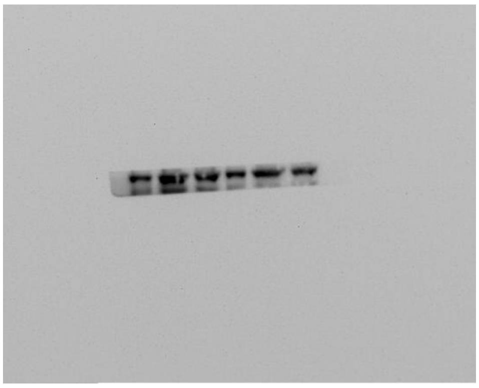

α-SMA

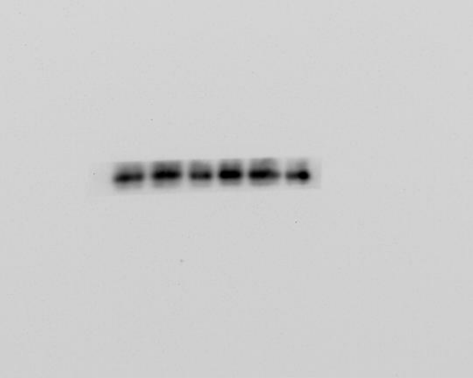

β-actin

Fig 3C

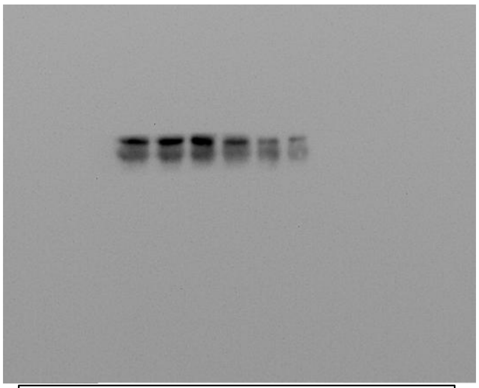

p-IGF1R(Thr1135/1136)

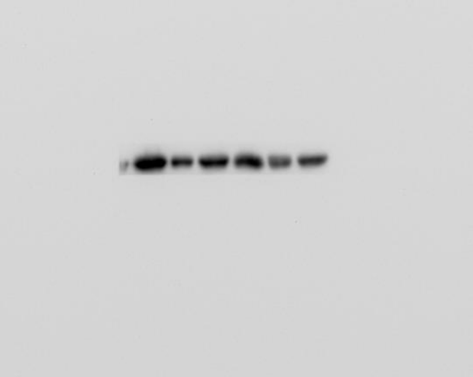

IGF1R

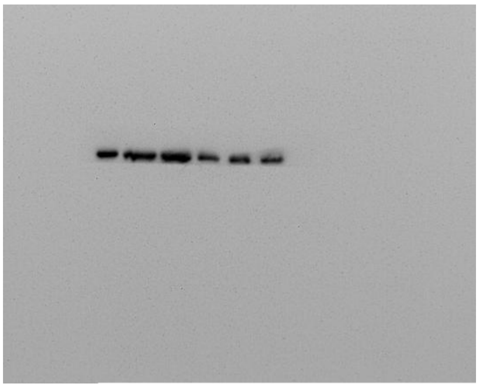

COL-1

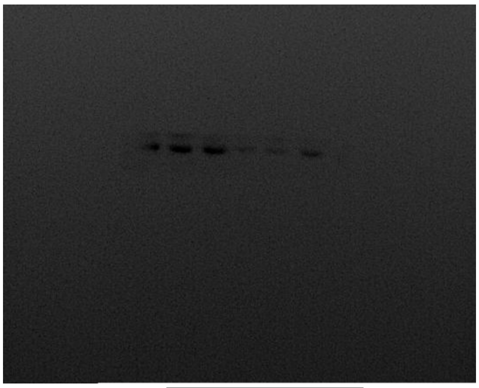

COL-3

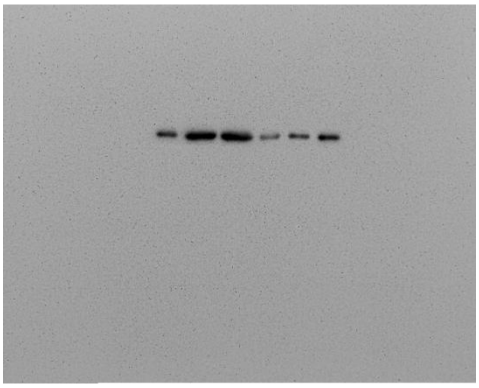

α-SMA

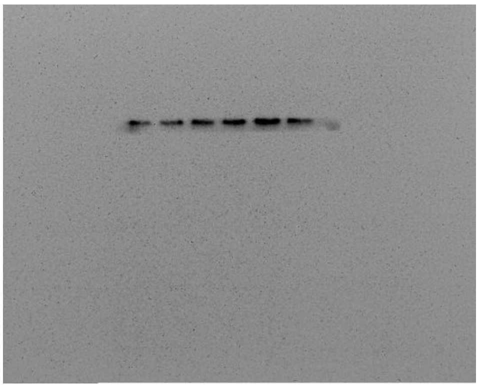

β-actin

Fig 3E

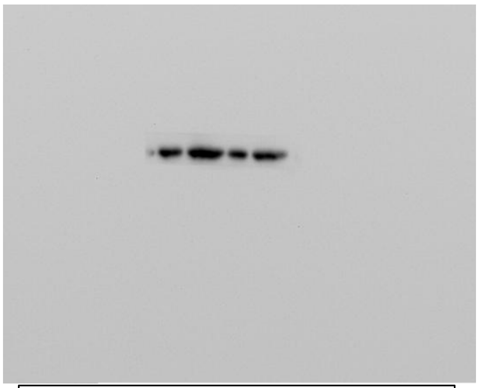

p-IGF1R(Thr1135/1136)

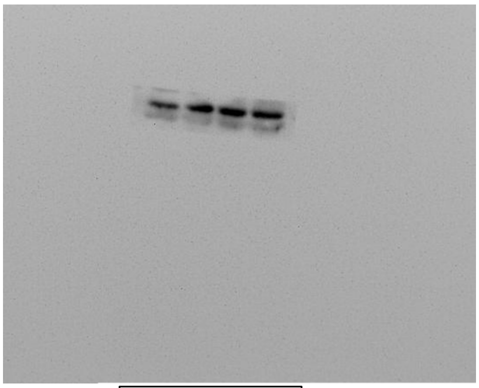

IGF1R

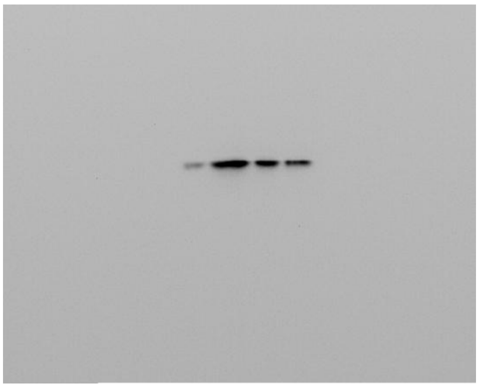

COL-1

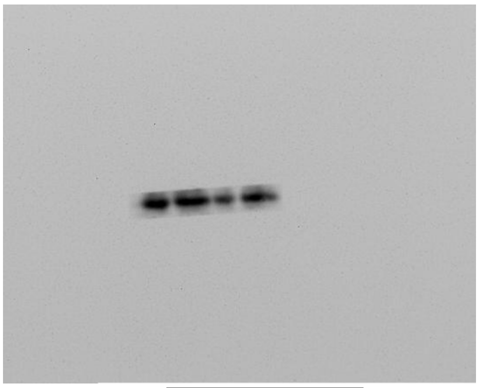

COL-3

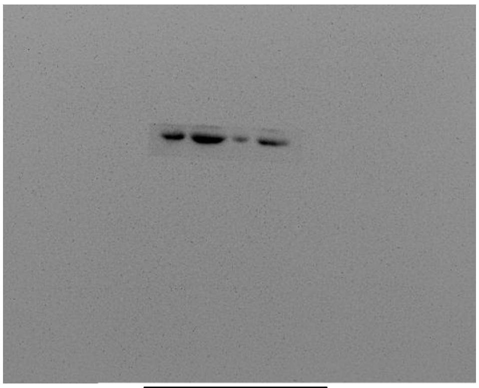

α-SMA

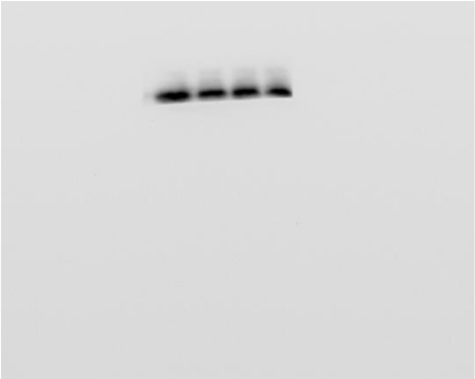

β-actin

Fig 4B

A549

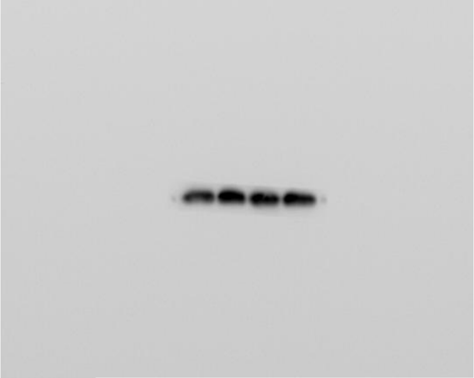

HO-1

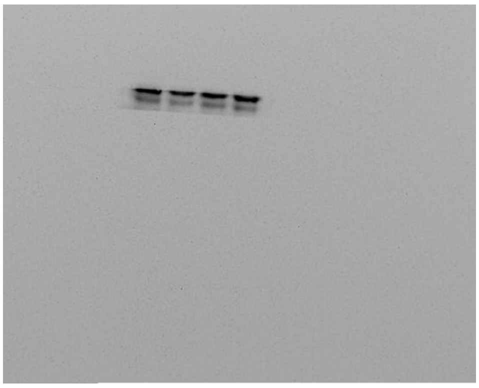

β-actin

BEAS-2B

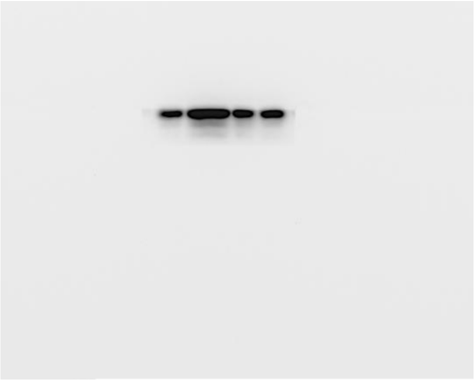

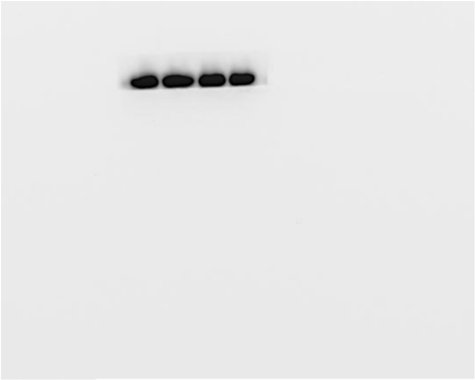

HO-1

β-actin

Fig 4D

A549

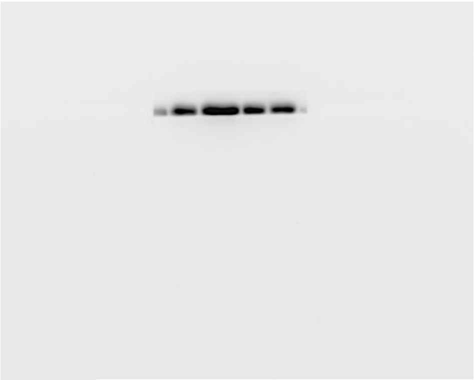

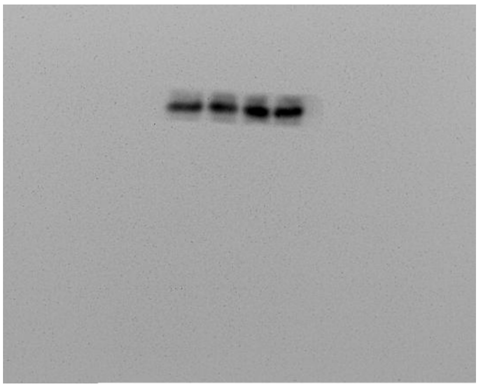

HO-1

β-actin

BEAS-2B

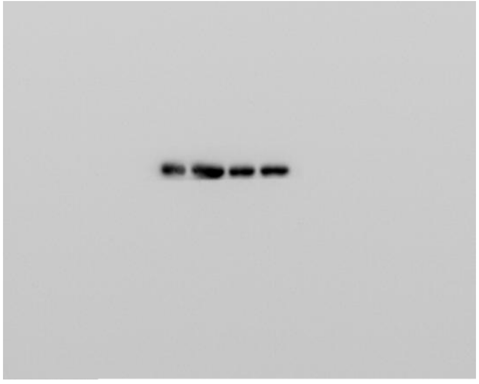

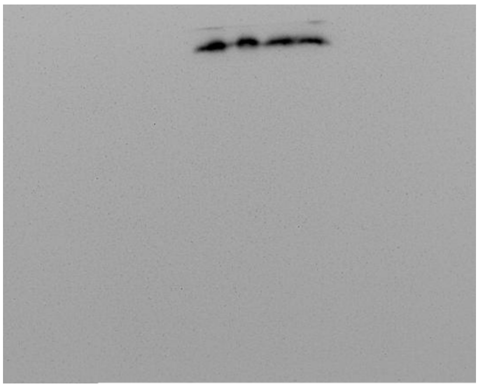

HO-1

β-actin

Fig 4F

A549-P40 A549

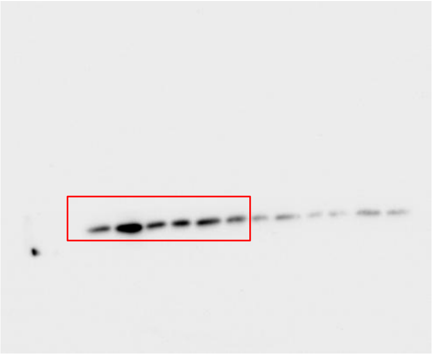

N-cadherin

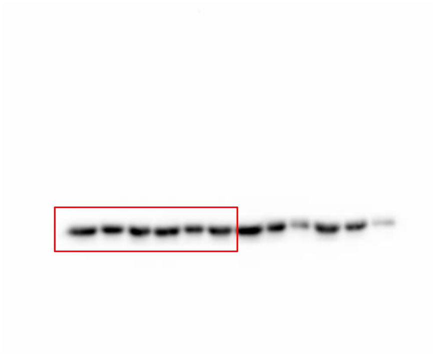

E-cadherin

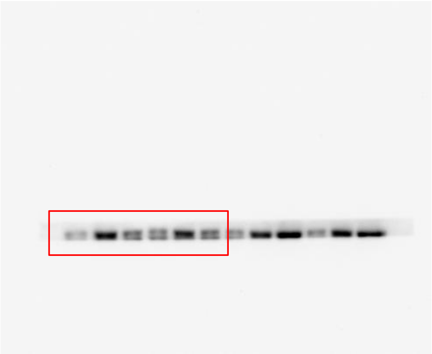

TGFβ1

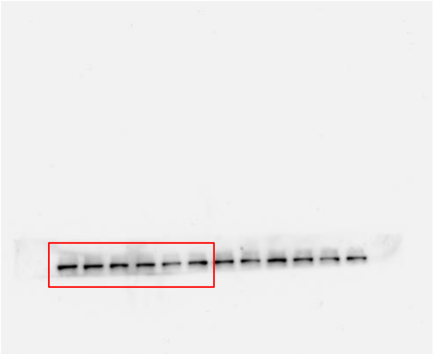

β-actin

BEAS-2B-P40 BEAS-2B

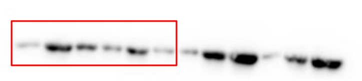

N-cadherin

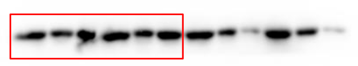

E-cadherin

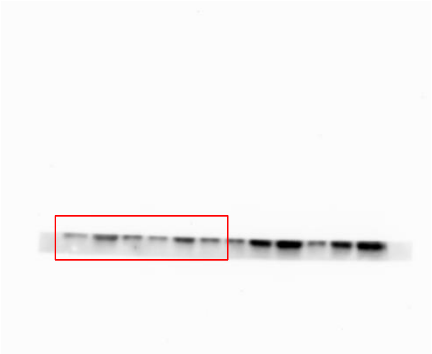

TGFβ1

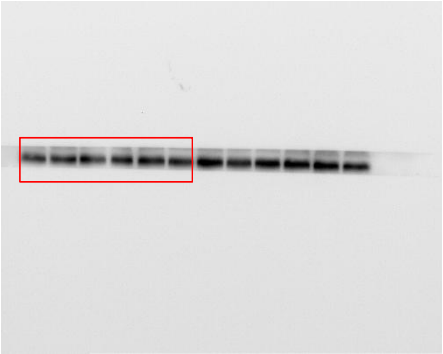

β-actin

Fig 6A

A549-P40 A549

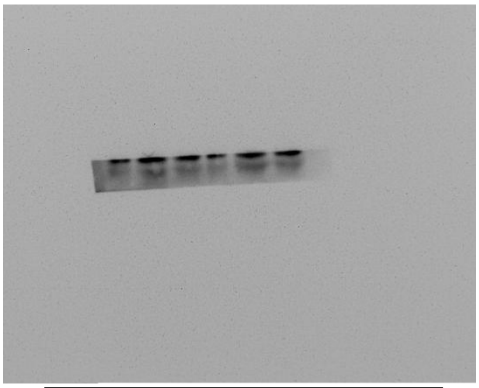

p-NF-κB p56(Ser536)

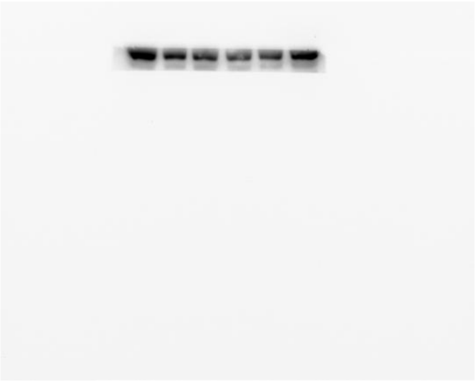

NF-κB p56

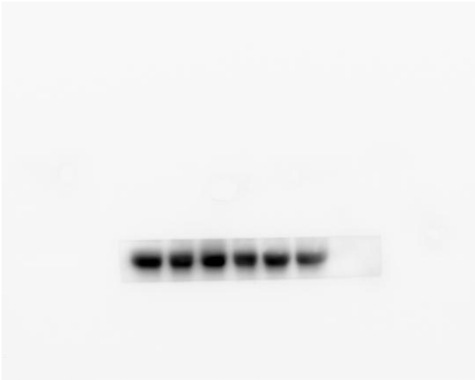

β-actin

BEAS-2B-P40 BEAS-2B

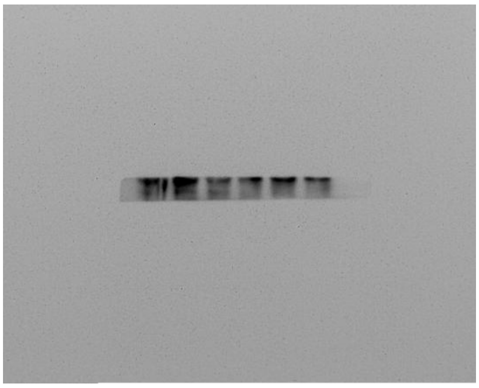

p-NF-κB p56(Ser536)

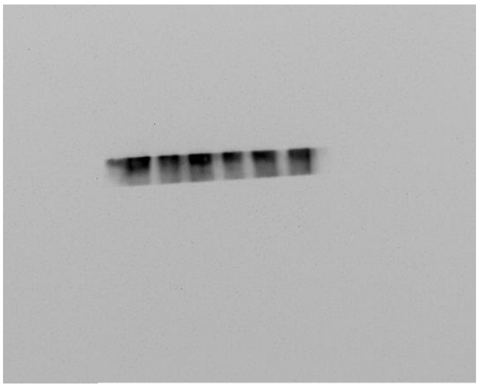

NF-κB p56

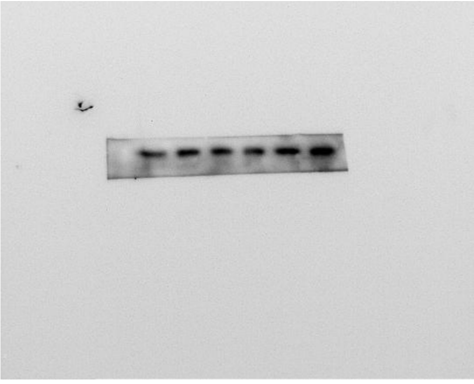

β-actin

Fig 6B

A549

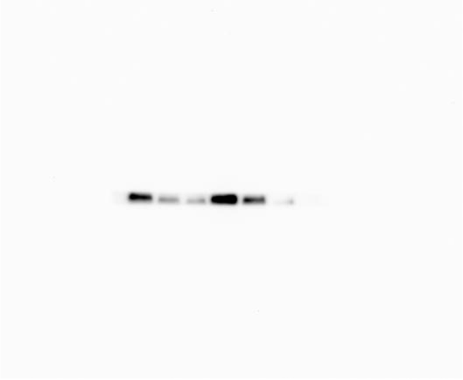

NLRP3

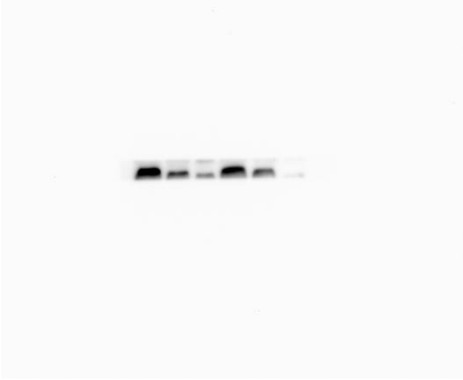

Cleaved Caspase1(Asp297)

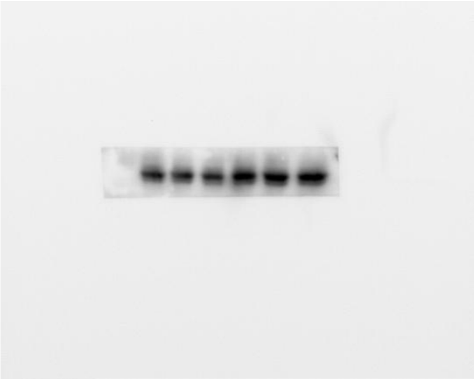

Caspase1

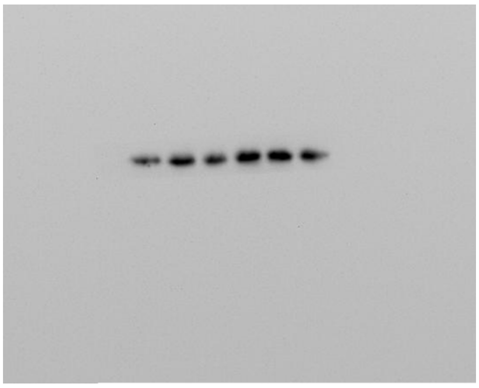

β-actin

BEAS-2B

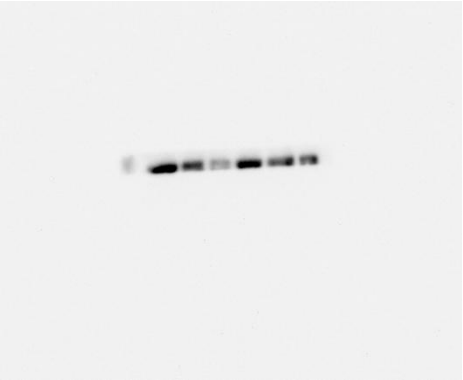

NLRP3

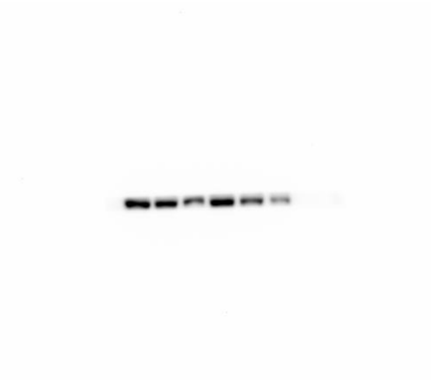

Cleaved Caspase1(Asp297)

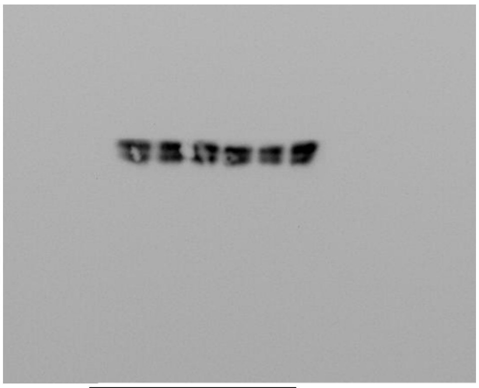

Caspase1

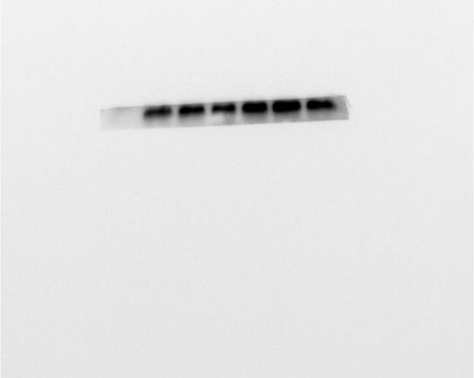

β-actin

Fig 6D

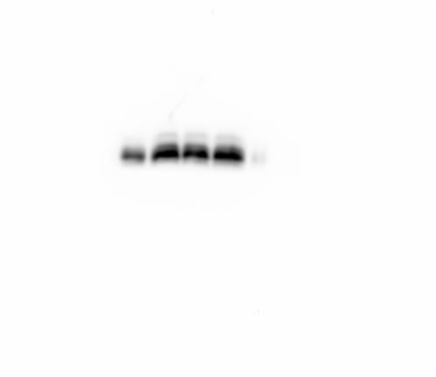

p-NF-κB p56(Ser536)

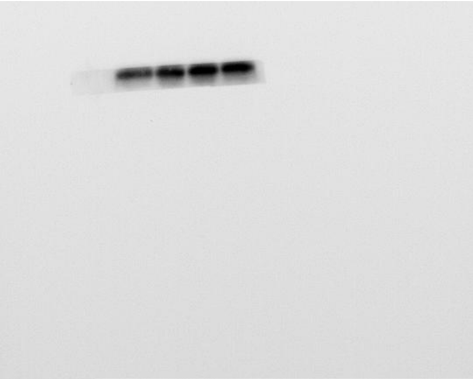

NF-κB p56

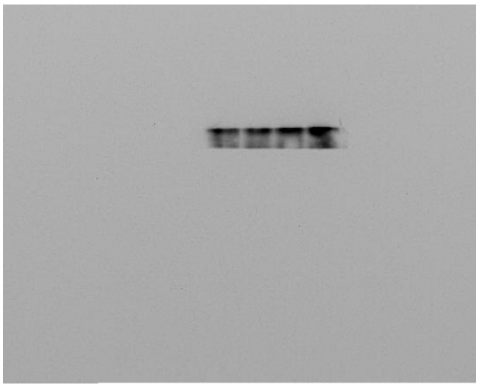

β-actin

Fig 6E

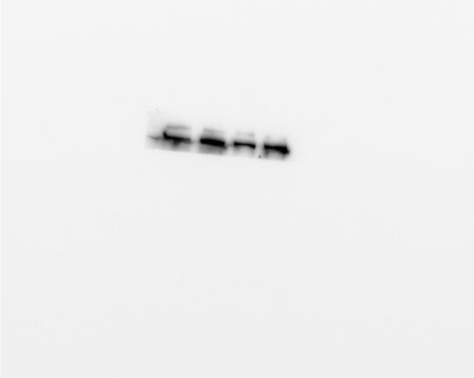

NLRP3

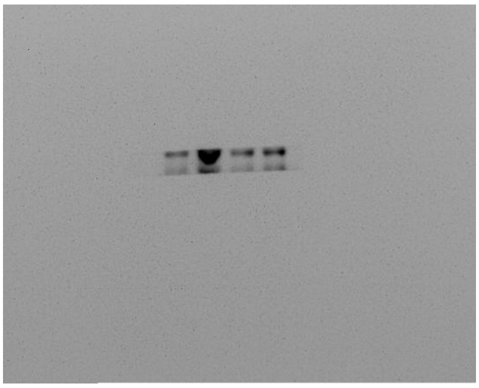

Cleaved Caspase1(Asp297)

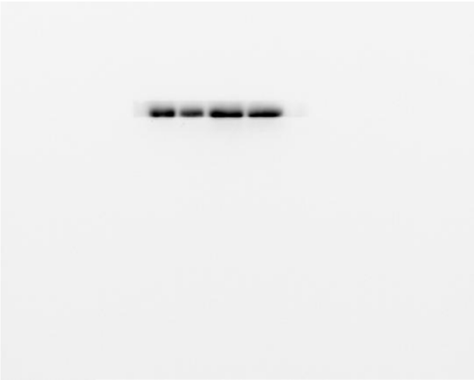

Caspase1

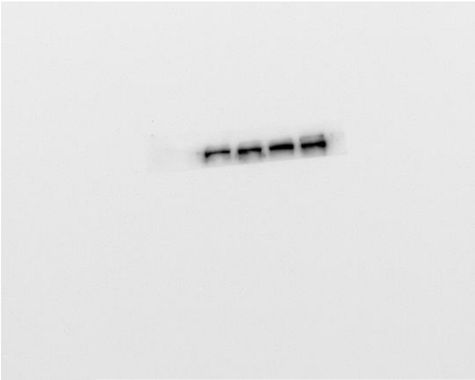

β-actin

Fig 7A

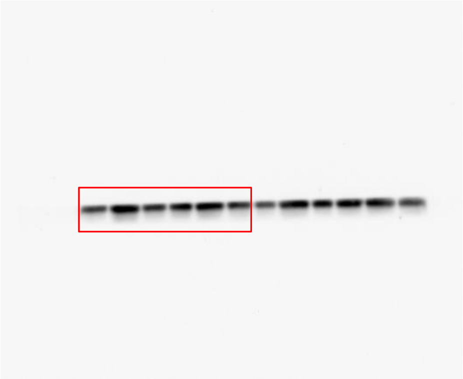

N-cadherin

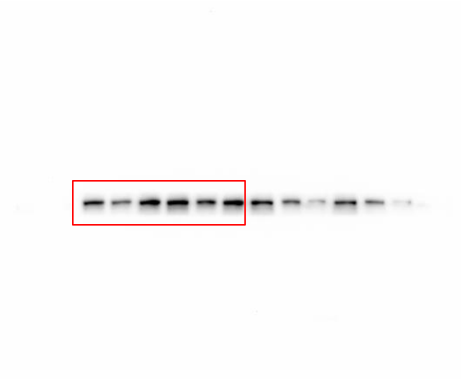

Ecadherin

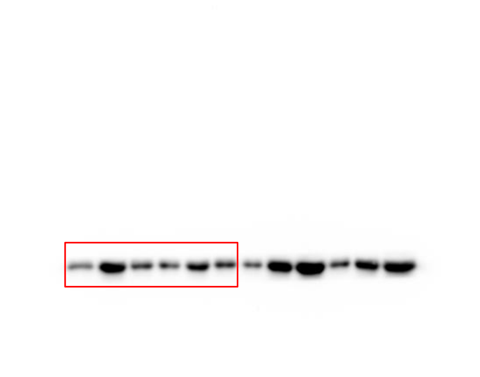

TGFβ1

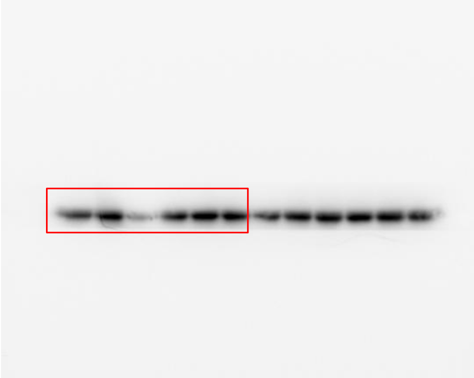

NLRP3

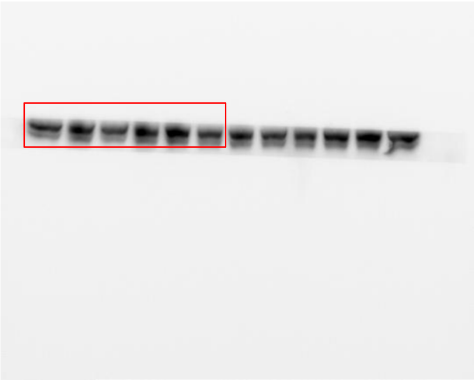

Cleaved Caspase1(Asp297)

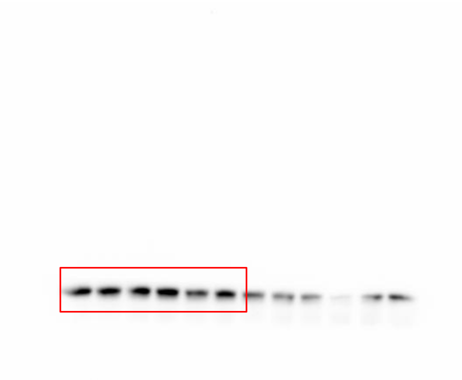

Caspase1

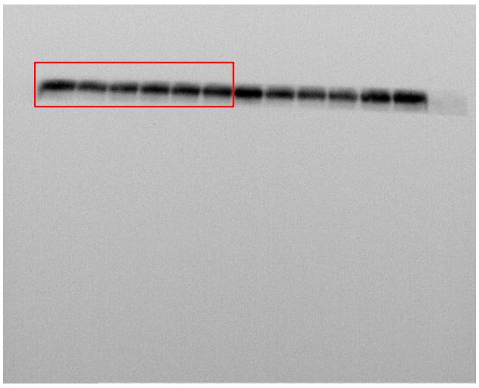

β-actin

Fig 8A

A549-P40 A549

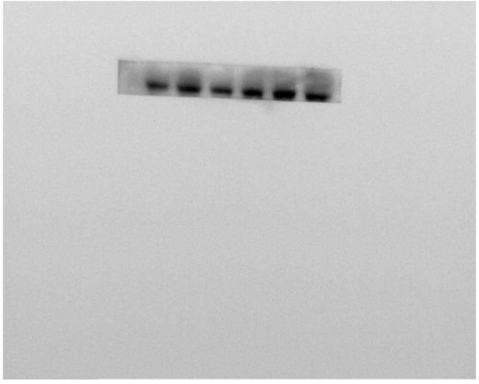

p-Akt(Ser473)

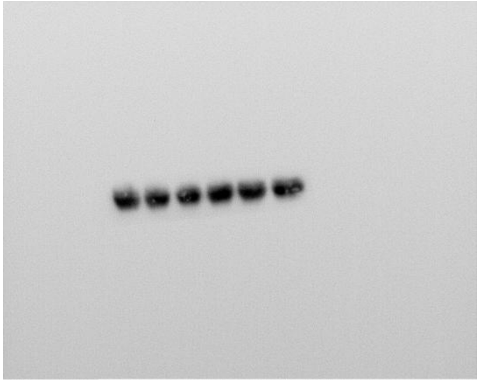

Akt

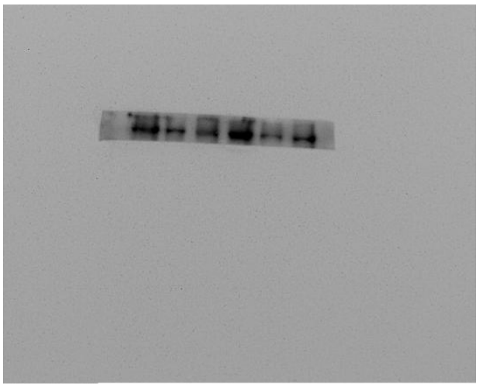

GSK3β

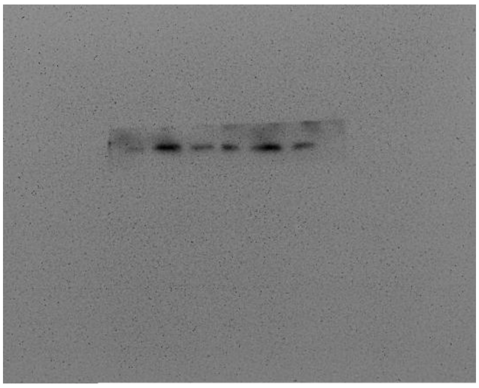

p-GSK3β(Ser9)

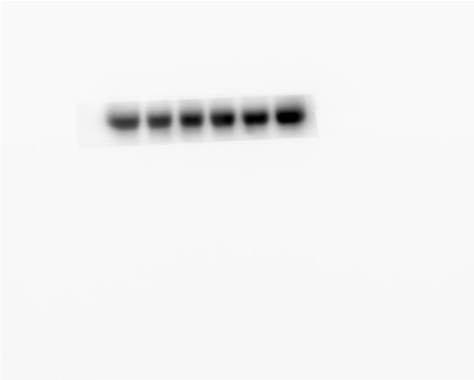

β-actin

BEAS-2B-P40 BEAS-2B

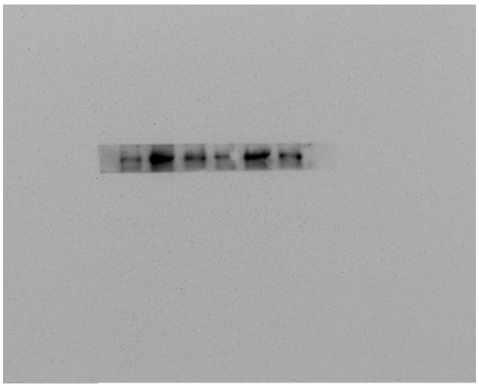

p-Akt(Ser473)

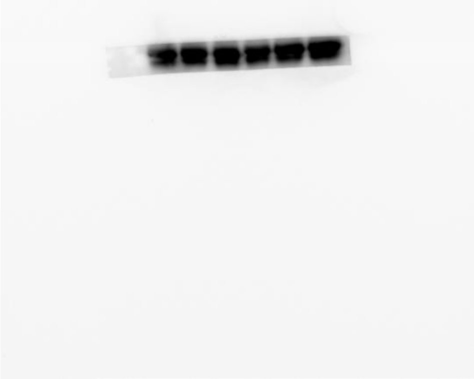

Akt

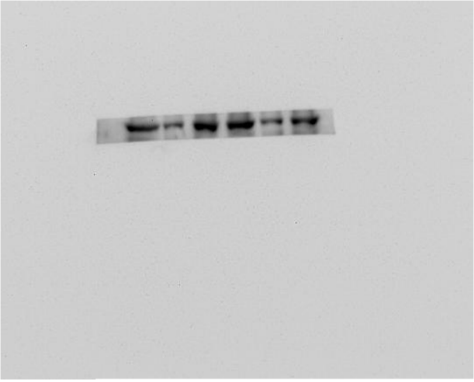

GSK3β

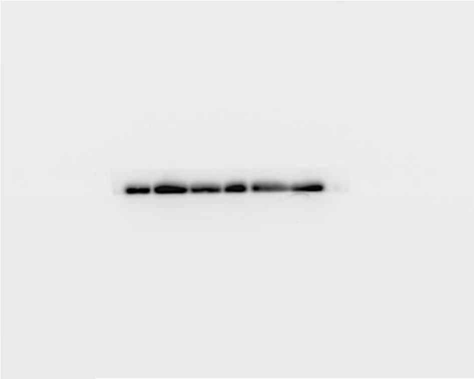

p-GSK3β(Ser9)

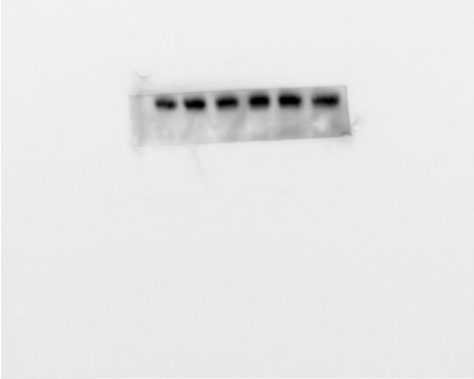

β-actin

Fig 8B

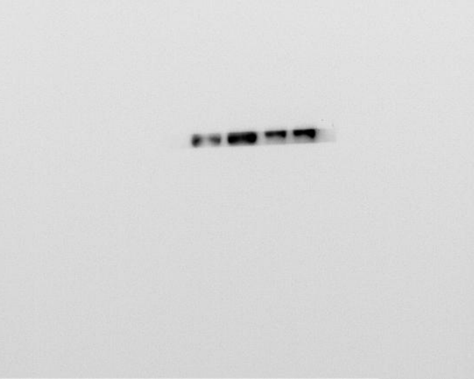

p-Akt(Ser473)

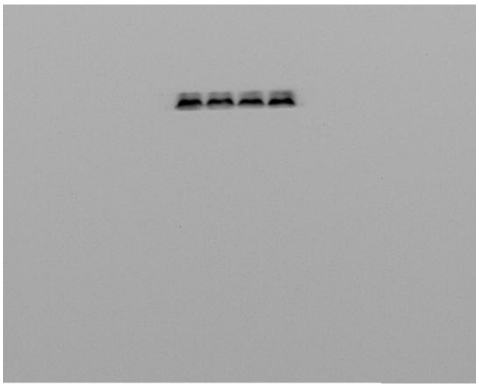

Akt

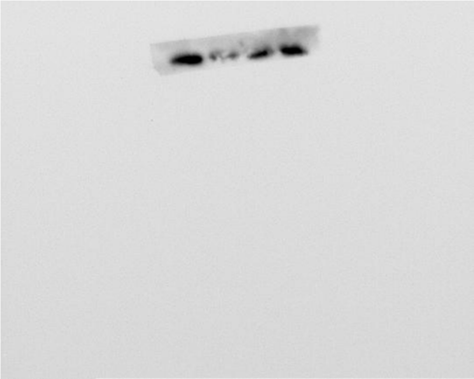

p-GSK3β(Ser9)

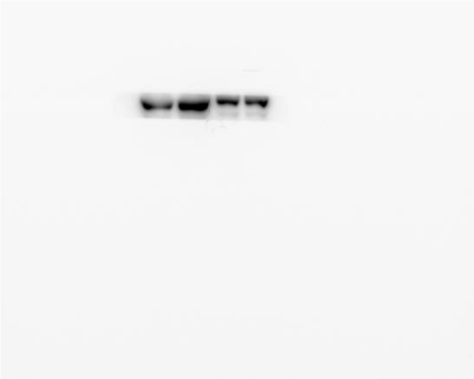

GSK3β

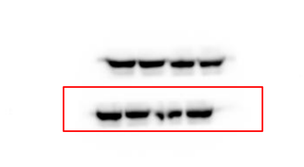

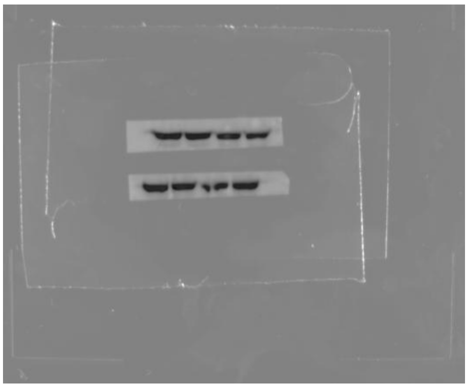

β-actin

Fig 8C

A549-P40

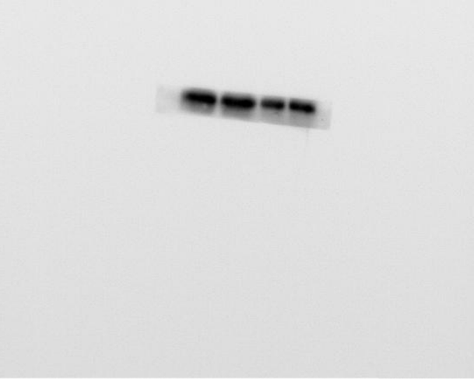

TGFβ1

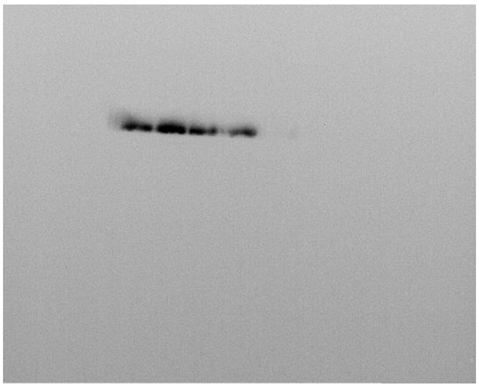

COL-1

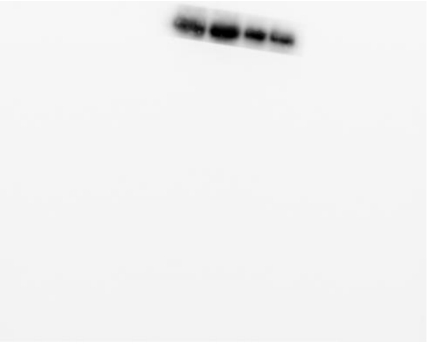

COL-3

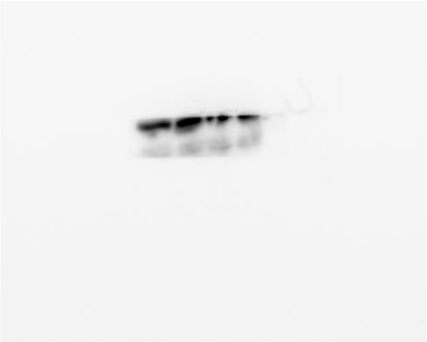

N-cadherin

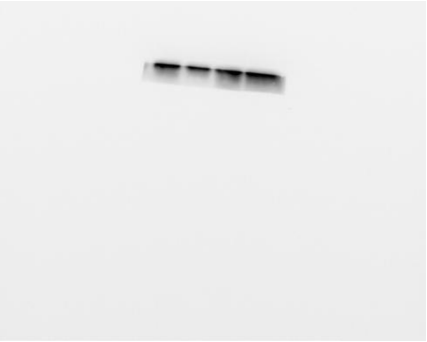

E-cadherin

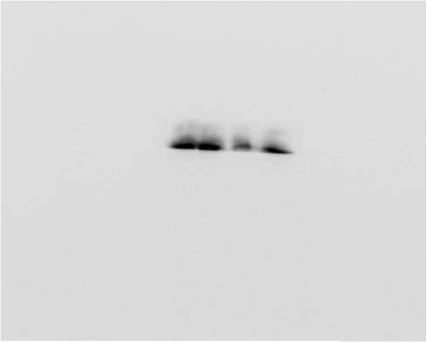

α-SMA

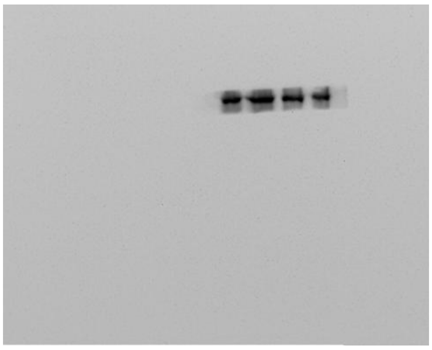

p-NF-κB p56(Ser536)

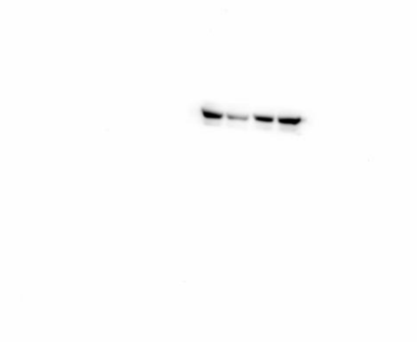

NF-κB p56

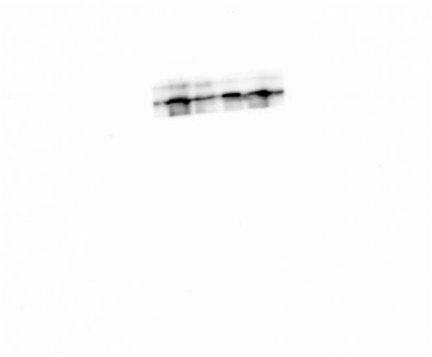

β-actin

BEAS-2B-P40

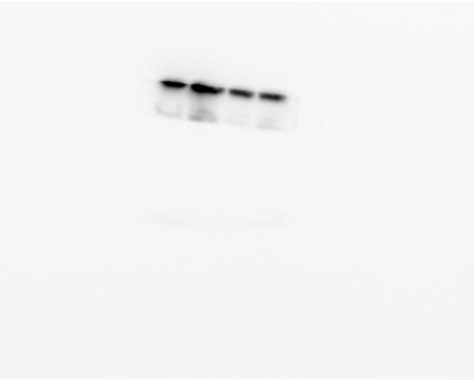

TGFβ1

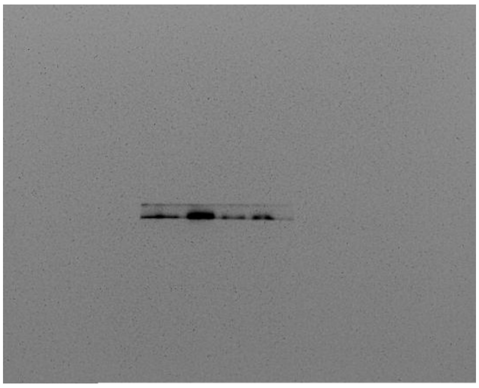

COL-1

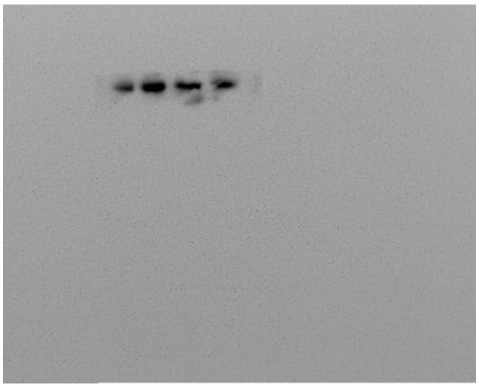

COL-3

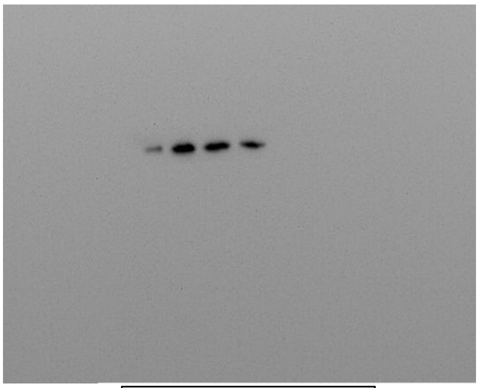

N-cadherin

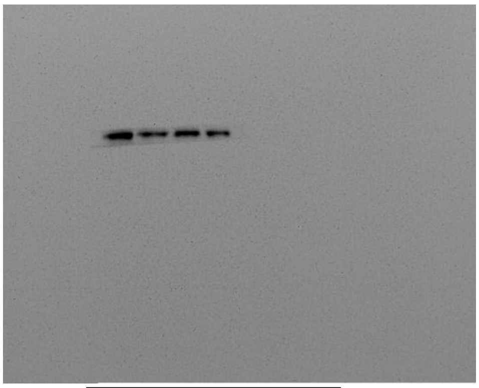

E-cadherin

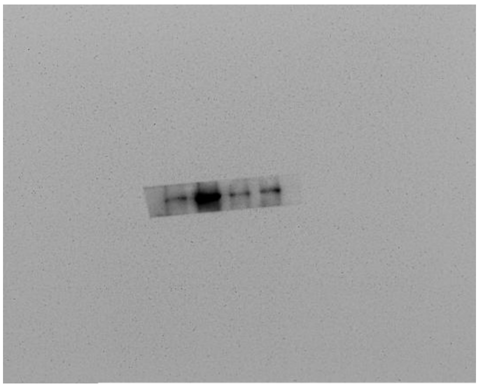

α-SMA

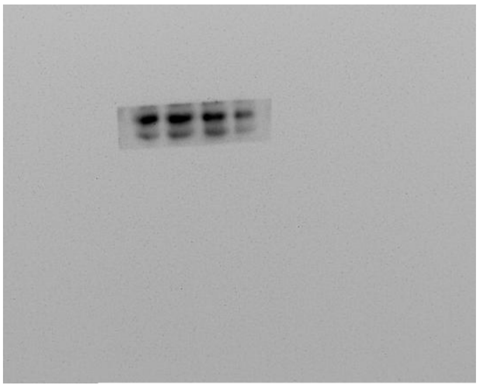

p-NF-κB p56(Ser536)

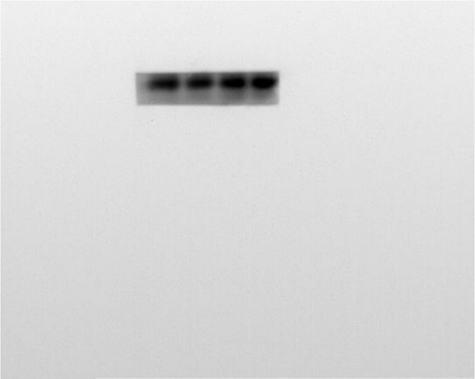

NF-κB p56

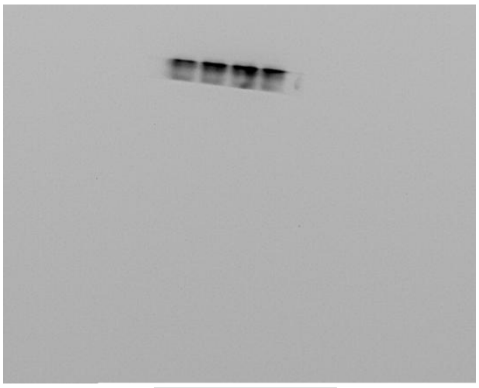

β-actin

Fig 8D

A549

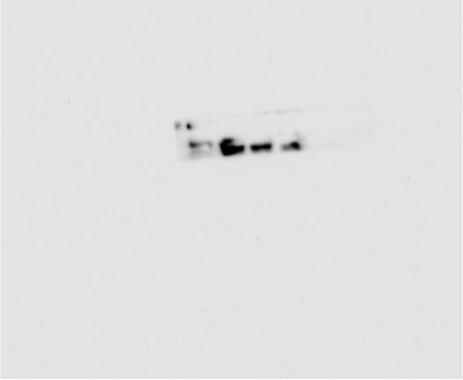

NLRP3

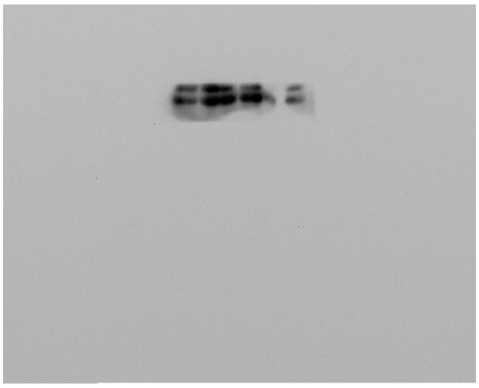

Cleaved Caspase1(Asp297)

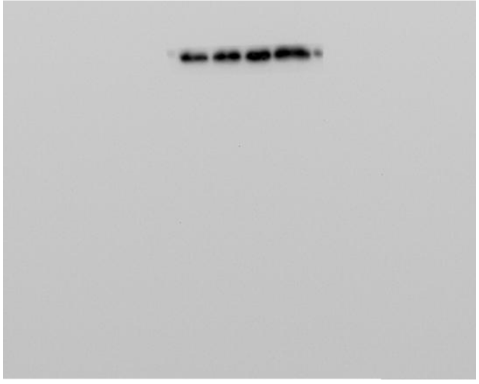

Caspase1

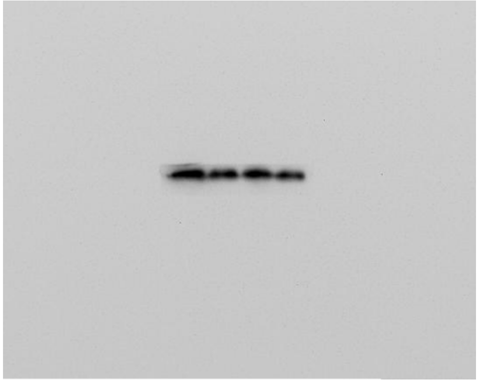

β-actin

BEAS-2B

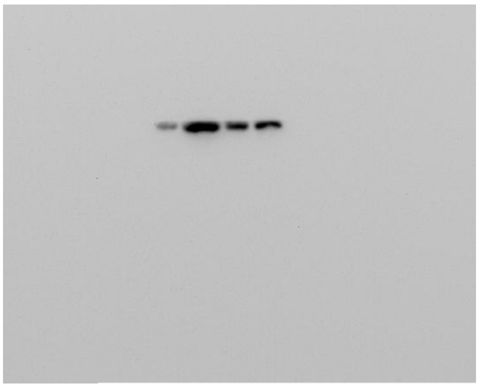

NLRP3

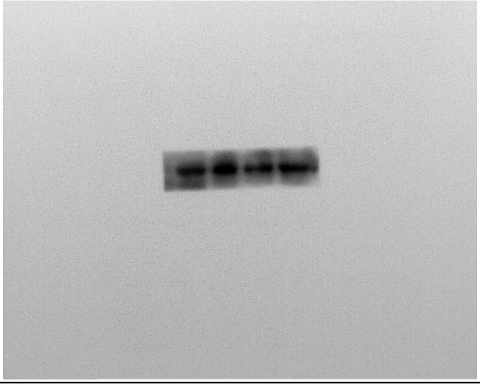

Cleaved Caspase1(Asp297)

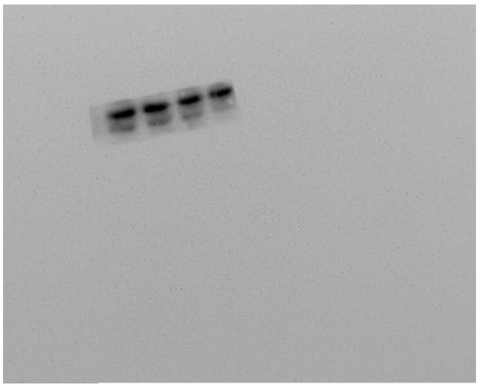

Caspase1

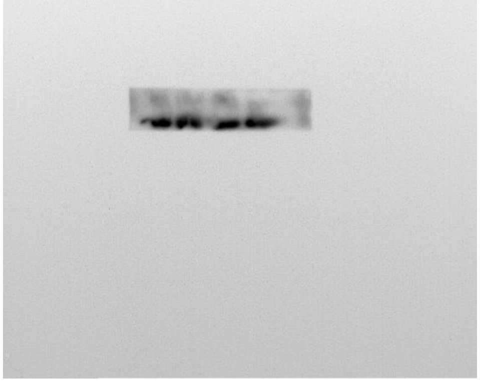

β-actin

Remark: Due to insufficient experimental funds, in the western blot assay, we cut the PVDF membrane into a membrane small enough to incubate the antibody according to the molecular weight of the incubated antibody and the protein molecular weight marker. We have made the above remark in the relevant legends of the manuscript.

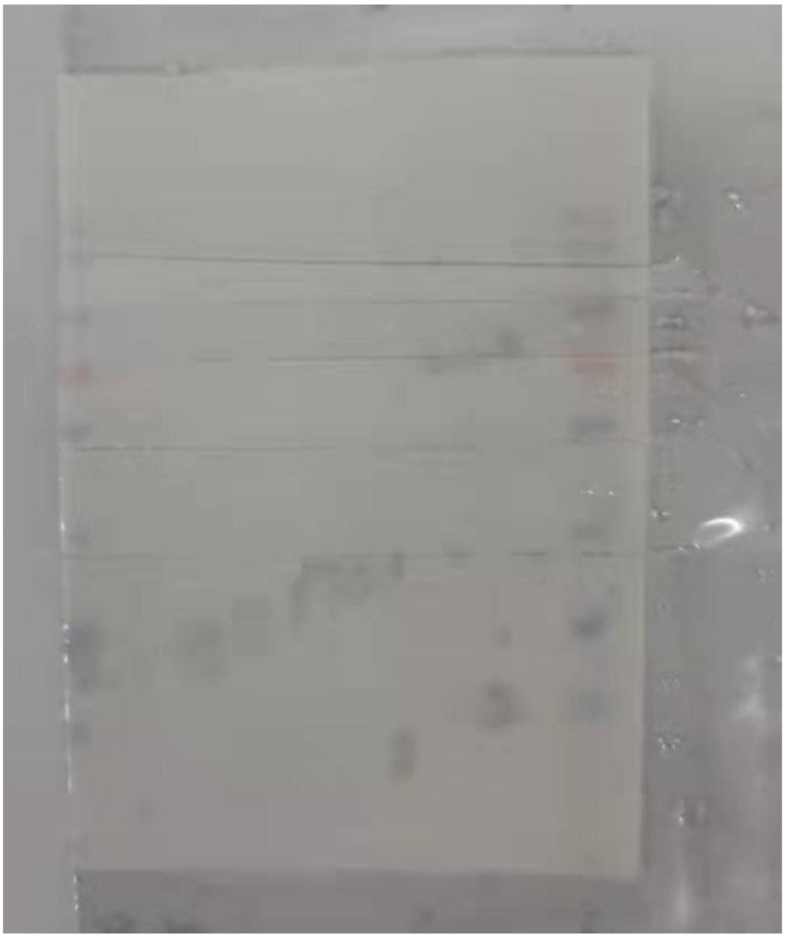

Supplement: Supplementary file 2 — Original Data File [file 41420_2022_1291_MOESM2_ESM.pdf]
